# Exploiting the interactions between the ruthenium Hoveyda–Grubbs catalyst and Al-modified mesoporous silica: the case of SBA15 *vs.* KCC-1†
†Electronic supplementary information (ESI) available. See DOI: 10.1039/c7sc05200f


**DOI:** 10.1039/c7sc05200f

**Published:** 2018-03-05

**Authors:** Baraa Werghi, Eva Pump, Mykyta Tretiakov, Edy Abou-Hamad, Andrei Gurinov, Pradeep Doggali, Dalaver H. Anjum, Luigi Cavallo, Anissa Bendjeriou-Sedjerari, Jean-Marie Basset

**Affiliations:** a KAUST Catalysis Center (KCC) , King Abdullah University of Science and Technology , Thuwal , 23955-6900 , Saudi Arabia . Email: jeanmarie.basset@kaust.edu.sa ; Email: Anissa.BendjeriouSedjerari@kaust.edu.sa; b King Abdullah University of Science and Technology (KAUST) , Core Labs , Thuwal , 23955-6900 , Saudi Arabia

## Abstract

Immobilization of the 2^nd^ generation Hoveyda–Grubbs catalyst **HG-II** onto well-ordered 2D hexagonal (SBA15) and 3D fibrous (KCC-1) mesostructured silica, which contained tetra-coordinated Al, has been investigated through the Surface Organometallic Chemistry (SOMC) methodology. The main interest of this study lies in the peculiarity of the silica supports, which display a well-defined tetrahedral aluminum hydride site displaying a strong Lewis acid character, [(≡Si–O–Si≡)(≡Si–O–)_2_Al–H]. The resulting supported Hoveyda–Grubbs catalysts have been fully characterized by advanced solid state characterization techniques (FT-IR, ^1^H and ^13^C solid state NMR, DNP-SENS, EF-TEM…). Together with DFT calculations, the immobilization of **HG-II** does not occur through the formation of a covalent bond between the complex and the Al-modified mesoporous silica as expected, but through an Al···Cl–[Ru]-coordination. It is not surprising that in functionalized olefin metathesis of diethyldiallyl malonate, DEDAM (liquid phase), leaching of the catalyst is observed which is not the case in non-functionalized olefin metathesis of propene (gas phase). Besides, the results obtained in propene metathesis with **HG-II** immobilized either on SBA15 (*d*_pore_ = 6 nm) or KCC-1 (*d*_pore_ = 4 or 8 nm) highlight the importance of the accessibility of the catalytic site. Therefore, we demonstrate that KCC-1 is a promising and suitable 3D mesoporous support to overcome the diffusion of reactants into the porous network of heterogeneous catalysts.

## Abstract 2

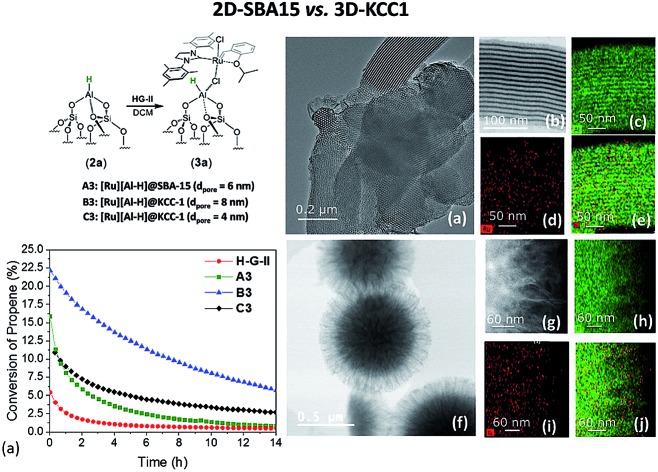
2^nd^ generation Hoveyda–Grubbs catalyst immobilized onto well-ordered 2D hexagonal (SBA15) and 3D fibrous (KCC-1) mesostructured silica displaying tetra-coordinated Al–H *via* Surface Organometallic Chemistry (SOMC).

## 1 Introduction

**Fig. 1:**
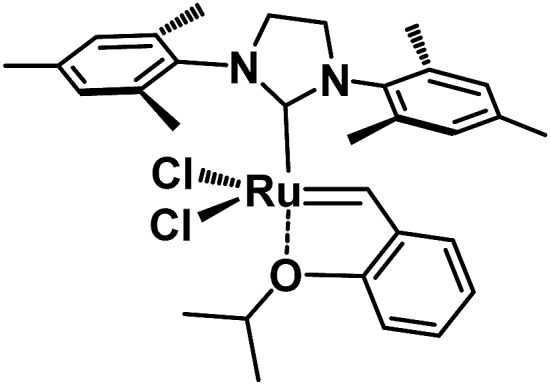
The 2^nd^ generation Hoveyda–Grubbs catalyst **HG-II**.

Regarding the development of homogeneous olefin metathesis, ruthenium(ii) catalysts have impacted on numerous applications ranging from industrial processes involving polymers, pharmaceuticals and fine chemicals.^1^–^5^ One of the most eminent homogeneous catalysts to perform olefin metathesis reactions is the second generation Hoveyda–Grubbs catalyst, **HG-II**,^6^ bearing a Ru(ii) metal centre surrounded by an N-heterocyclic carbene (NHC), two anionic chlorine ligands and one chelating benzylidene ligand containing an ether functionality coordinated to Ru (Fig. 1).^1^,^7^,^8^

This catalytic system is particularly efficient for metathesis reactions involving highly electron-deficient substrates. The most significant breakthrough of the **HG-II** catalyst is its tolerance to functional groups and therefore its ability to perform the metathesis of functional olefins,^9^ ring closing metathesis (RCM), ring opening metathesis polymerization (ROMP) and cross metathesis (CM).^3^,^10^–^15^ Despite its impressive catalytic activity, versatility and stability, shortcomings still need to be resolved including easy separation from the reaction medium, recyclability and bimolecular decomposition of the homogeneous catalyst.^16^,^17^ These issues can be overcome by immobilization of **HG-II** on solid supports. To date, many attempts using the supported homogeneous catalysis strategy over hybrid mesoporous silica have been made,^18^–^21^ but this approach leads to ill-defined supported homogeneous catalysts which are different from heterogeneous catalysts as the complexes may interact further with the surface.^22^–^25^ Recently, some groups demonstrated that a simple adsorption of **HG-II** onto different mesoporous silicas (SBA15, MCM41, SBA1…) induces a confinement of the Ru complex (size of **HG-II**: 1.76 × 1.37 × 1.047 nm^3^)^26^ inside the mesopores (from 1.5 to 6 nm) and therefore enables recyclability of the solid catalyst for olefin metathesis.^26^–^28^ In this case, the polarity of the solvent is crucial; lower polarity is preferred to avoid leaching of the Ru complex from the solid. However, the type of interaction between the Ru catalyst and the silica support remains unresolved, preventing the establishment of a clear anchoring mechanism of the complex onto the support. As a consequence, one would wonder whether the silica supported Ru retains its own activity and if the catalyst acts as a true heterogeneous catalyst.

The Surface Organometallic Chemistry (SOMC) methodology is a well-established approach to design well-defined single site supported catalysts featuring truly heterogeneous activity.^29^ The strategy is based on the reaction of a given organometallic compound (*e.g*. WMe_6_, TaMe_5_…) with isolated silanol of highly dehydroxylated silica and leads to the formation of a surface organometallic fragment (SOMF).^29^,^30^ However, the design of well-defined and single site ruthenium based SOMFs is difficult mainly due to the low affinity of Ru(ii) complexes towards oxygen containing ligands. Recently, we succeeded in developing a novel type of chemically modified support for SOMC applications.^31^–^33^ Among them, a well-defined tetrahedral aluminum hydride site, [(≡Si–O–Si≡)(≡Si–O–)_2_Al–H], [Al–H], was synthesized by reaction of di-isobutyl aluminum hydride (DIBAL) with the isolated silanol of a dehydroxylated SBA15 (700 °C, 10^–5^ mbar).^34^

In this work, we were interested to study the type of interaction between **HG-II** and the supported Lewis acid [Al–H] site grafted onto two types of modified mesoporous silica, SBA15 and KCC-1. Combining advanced solid state spectroscopies (FT-IR, SS NMR, DNP-SENS, EF-TEM…) and DFT calculations, we were able to propose an anchoring mechanism between **HG-II** and the Al-modified mesoporous silica. We will show that an Al···Cl–[Ru] interaction is responsible for the immobilization of **HG-II**. In parallel, we will demonstrate how the mesostructure of the silica (2D *vs.* 3D and pore diameter) affects the catalytic activity in propene metathesis.

## 2 Results and discussion

### Generation of [Al–H] surface groups on well-ordered mesoporous silica SBA15 and KCC-1

Two types of mesoporous materials were chosen: SBA15 (*d*_pore_ = 6 nm)^35^ and KCC-1 (*d*_pore_ = 8 and 4 nm).^36^,^37^ SBA15 is one of the most used mesoporous silicas in the field of heterogeneous catalysis. It was chosen because of its following structural parameters: 2D well-ordered hexagonal mesostructure, high surface area (800 m^2^ g^–1^), large uniform pore diameter (6 nm), and thermal (up to 1200 °C) and mechanical stability.^35^ However, it has been demonstrated that the accessibility of the active site and/or a hindered diffusion (of the substrate and products) inside the mesopores remains an important issue which needs to be resolved. To overcome these limitations, 3D well-ordered mesoporous materials appear to be the ideal candidates as these supports avoid diffusion issues and provide better active site accessibility. In 2010, our group developed a new family of high-surface area silica nano-spheres, KCC-1, with a spectacular 3D fibrous morphology combined with high surface area (>600 m^2^ g^–1^), high range of particle size (170–1120 nm),^37^ and high thermal, chemical and mechanical stability. The structural parameters, surface area and pore size of both parent materials, SBA15 and KCC-1, are given in the ESI (Fig. S7–S9, ESI).† All materials exhibit a well-ordered mesoporous structure according to their nitrogen sorption isotherms. Transmission Electron Microscopy (TEM) clearly shows a 2D hexagonal structure for SBA15 (Fig. 6a) while KCC-1 is characterized by a 3D fibrous morphology (Fig. 6f). Hence, the morphology of the materials, their structure and hierarchical organization (2D or 3D networks, shape…) might affect the catalytic activity based on the accessibility of the active sites.

The generation of well-defined tetrahedral aluminum hydride [Al–H] on SBA15_700_ (*d*_pore_ = 6 nm), **A0**, KCC-1_700_ (*d*_pore_ = 8 nm), **B0**, and KCC-1_700_ (*d*_pore_ = 4 nm), **C0**, was achieved as previously described in the literature.^34^,^36^,^37^ It consists of a dehydroxylation pretreatment of mesoporous silica (700 °C, 10^–5^ mbar, 30 h), which leads to the formation of isolated silanol (≡SiOH) (ESI†).

The reaction of dehydroxylated mesoporous silica **A0**, **B0** and **C0** with DIBAL (1 eq. per [≡SiOH]) leads to a bipodal well-defined single-site tetrahedral iso-butylaluminum supported complex, [(≡Si–O–Si≡)(≡Si–O–)_2_Al–^*i*^Bu], **1a**, along with silicon hydride, [≡Si–H], **1b**, and silicon isobutyl, [≡Si–CH_2_CH(CH_3_)_2_], **1c**.

**Scheme 1:**
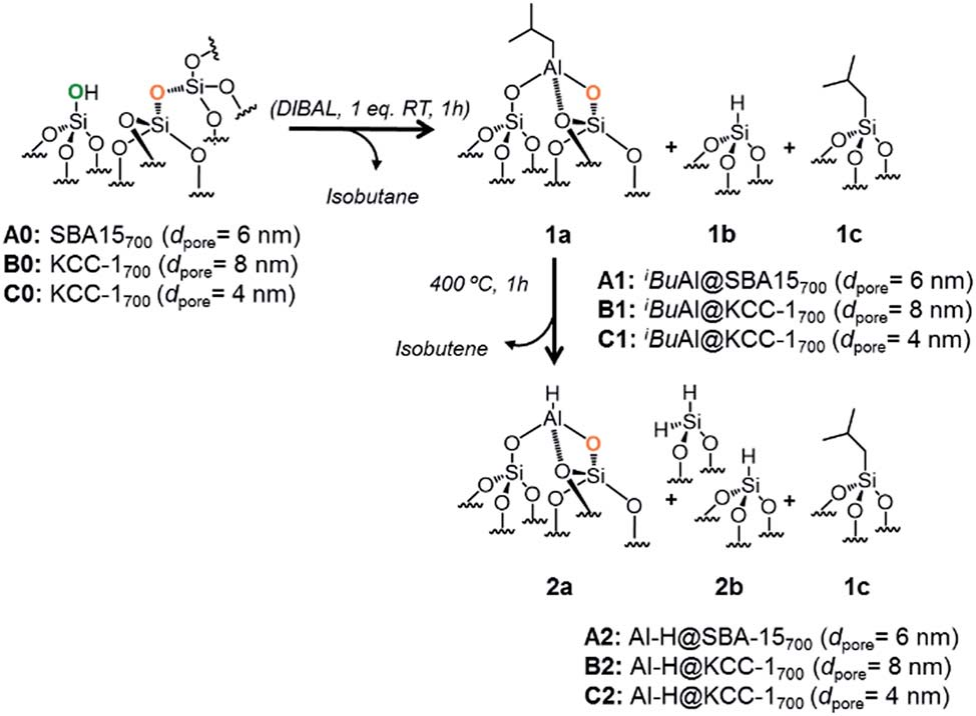
Reaction of DIBAL with **A0**, **B0** and **C0** (1 eq. DIBAL/silanol) to yield **A1**, **B1** and **C1** followed by thermal treatment leading to **A2**, **B2** and **C2**.

[Al–H], **2a**, the hydride homologue of **1a**, is obtained by a simple thermal treatment (ESI†) obtained through a β-H elimination from the –CH_3_ of the isobutyl moiety to the Al-center (Scheme 1). Advanced solid state characterization (^1^H, ^13^C, ^27^Al and ^29^Si SS NMR and FT-IR) and DFT calculations provided clear knowledge of the atomic composition of the surface site, which is essential to establishing structure–activity relationships at the molecular and atomic levels.^33^,^34^

**Fig. 2:**
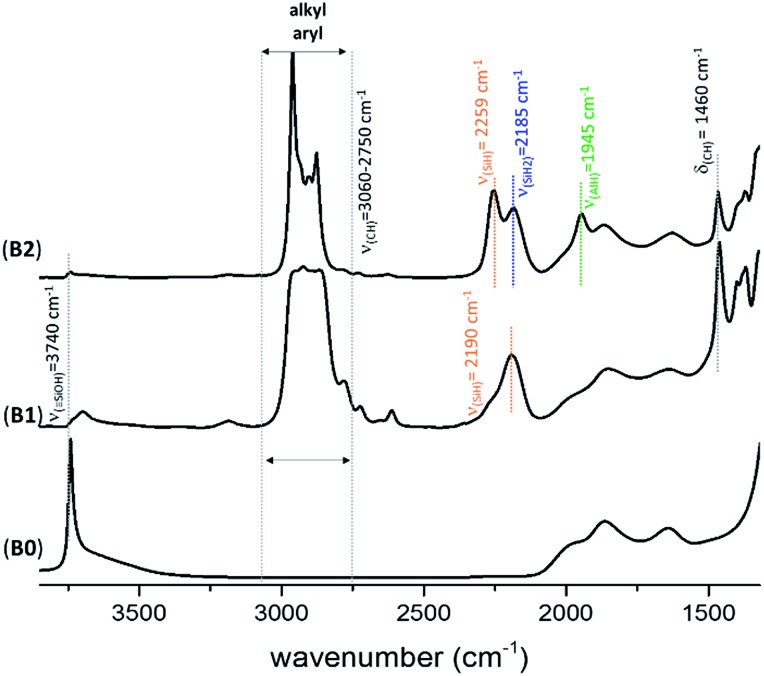
FT-IR spectra of KCC-1_700_ (**B0**), Al^*i*^Bu@KCC-1_700_ (**B1**) and Al–H@KCC-1_700_ (**B2**).

The FT-IR spectra of KCC-1 **B2** (Fig. 2) and **C2** (Fig. S4, ESI†) are similar to the one previously obtained with SBA15 **A2** (Fig. S1, ESI†). Fig. 2 shows the FT-IR spectrum of **B0**, **B1** and **B2** after each step of synthesis (dehydroxylation at 700 °C for 30 h, reaction with DIBAL for 1 h and thermal treatment at 400 °C for 1 h, respectively). The characteristic vibrational band of isolated silanol, *ν*(OH) at 3747 cm^–1^, instantaneously disappears after reaction with DIBAL (**B1**, Fig. 2). Meanwhile, the alkyl vibrational bands *ν*(CH) of [(≡Si–O–Si≡)(≡Si–O–)_2_Al–^*i*^Bu] and [≡Si–^*i*^Bu] appear from 3060 to 2750 cm^–1^. The FT-IR band at 2190 cm^–1^*ν*(Si–H) is assigned to silicon monohydride.^31^,^33^,^34^

The generation of the terminal [(≡Si–O–Si≡)(≡Si–O–)_2_Al–*H*] is characterized by the presence of the band *ν*(Al–H) at 1945 cm^–1^. The vibrational bands at 2259 and 2185 cm^–1^ are characteristic of *ν*(SiH) and *ν*(SiH_2_), respectively.^33^,^34^ Moreover, the alkyl vibrational bands of (≡Si–^*i*^Bu) in the region between *ν*(CH) = 3024 and 2823 cm^–1^ and the corresponding stretching bands *ν*(CH) = 1460 cm^–1^ and 1380 cm^–1^ maintain their respective frequency and intensity, showing that these alkyl groups remain on the surface.

Interestingly, the [Al–H] surface groups have been identified as strong Lewis acid sites through the adsorption/desorption of pyridine, p*K*_b_ = 5.21 (ESI†). Indeed, upon exposure to pyridine followed by evacuation at 400 °C at 10^–5^ mbar, the FT-IR spectrum displays three vibrational bands at 1455, 1578 and 1622 cm^–1^. These bands are assigned to the interaction between the lone pair of pyridine and the vacant orbital of Lewis acid sites, [Al–H] (Fig. S2, ESI†). We demonstrated by DFT calculations that the coordination of pyridine is favoured by –12.8 kcal mol^–1^. The aluminium orbital is accessible for the doublet of the nitrogen atom of pyridine as the coordination bond between the siloxane bridge [(≡Si–O–Si≡)] and the Al–H group from [(≡Si–O–Si≡)(≡Si–O)_2_ Al–H] is released, which is shown by an increased Al···O distance from 2.02 Å to 3.22 Å.

### Immobilization of the second generation Hoveyda–Grubbs ruthenium catalyst onto [Al–H] modified SBA15 and KCC-1

Reactions of complex **HG-II** with **A2**, **B2** and **C2** were conducted at room temperature in dichloromethane (DCM) for 5 h (ESI†). The amount introduced was 0.2 eq. [Ru]/[Al–H]. Above this value, the grafting is not complete. During the reaction, we clearly observe a colour change of the solution (from green to colourless) and the resulting materials **A3**, **B3** and **C3** become brown. Previously, it was mentioned that a reaction of **HG-II** with alumina might result in an immediate decomposition of the complex induced through Lewis acidic sites.^28^ This hypothesis could be disproved through the following study of **A3**, **B3** and **C3** by FT-IR, SS NMR, and DNP SENS characterization and catalytic tests. During the reaction of SBA15_700_, **A0** (blank experiment), with **HG-II**, the solution remains green and the materials turn light green.

The FT-IR spectra of **A3**, **B3** and **C3** (Fig. S3, 3 and S4,† respectively) show the appearance of new alkyl and aryl bands in the region between 3050 and 2800 cm^–1^ and 1608 cm^–1^, and their stretching bands *δ*(CH) at 1461 cm^–1^ and 1380 cm^–1^. The appearance of a small shoulder at 3070 cm^–1^, assigned to *ν*(C=C), together with another *δ*(C=C) stretching band at 1635 cm^–1^ suggests the presence of a C=C double bond. Significant changes involve the [Al–H] site, **2a**, which exhibits, according to DFT (but not observed experimentally because it is hidden in the ≡Si–O–Si≡ combination and overtone bands at 1639, 1864, 1973 cm^–1^), a red-shift from *ν*(Al–H) = 1947 cm^–1^ to 1893 cm^–1^ (Fig. S5, ESI†). Further, a shift of (≡Si–H) from *ν*(Si–H) = 2256 cm^–1^ to 2225 cm^–1^ and (=Si–H_2_) *ν*(SiH_2_) from 2191 cm^–1^ to 2144 cm^–1^ was observed, which is in accordance with the predicted IR spectrum from DFT calculations (Fig. S5, ESI†). A detailed explanation is given in the ESI.†

**Fig. 3:**
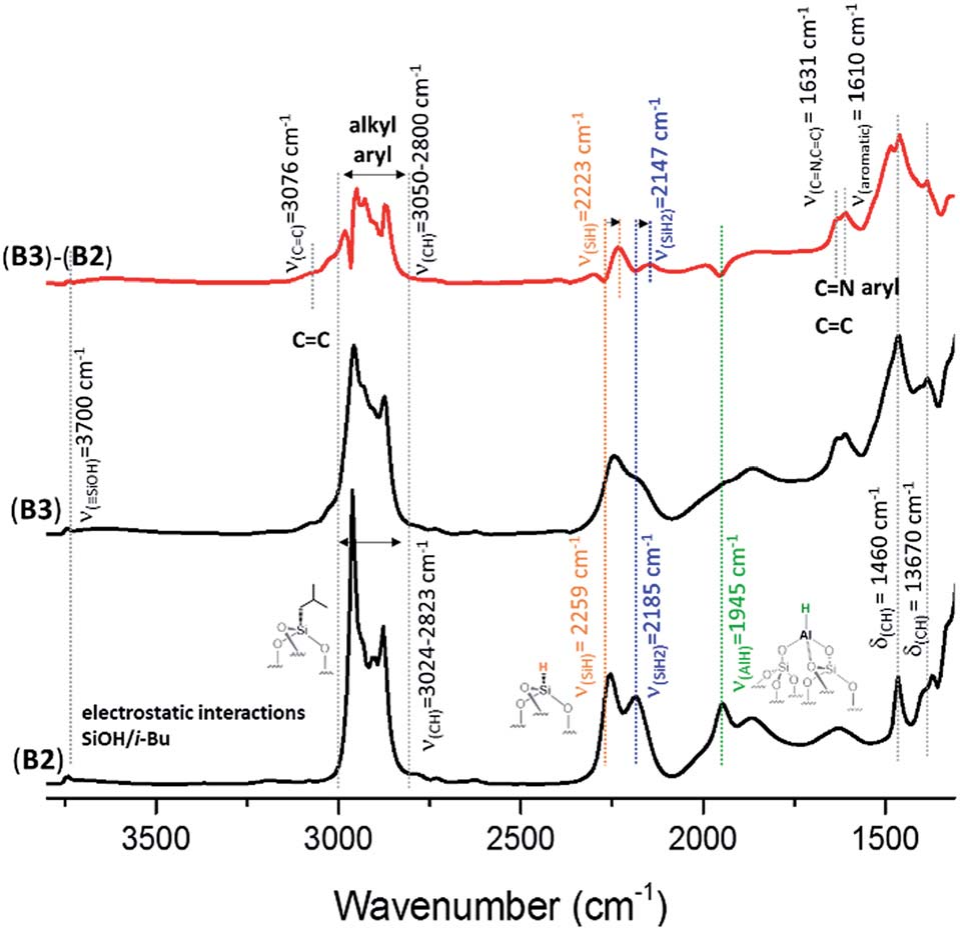
FT-IR spectra of Al–H@KCC-1_700_ (**B2**) and **B3** formed after reaction with **HG-II**. Subtraction (**B3 – B2**) is given in red.

After completion of the reaction, no gases (propane, propylene, or HCl) were released (ESI†). Elemental analysis (ESI†) shows the presence of 1.5 wt% of ruthenium on SBA15 (**A3**) and on KCC-1 (**B3**, *d*_pore_ = 8 nm) corresponding to a Ru/Al-ratio of 0.1 and hence to a consumption of 10–15% of the available Al–H sites. The results are in agreement with energy dispersive X-ray (EDS) spectra measured by TEM for the regions shown in Fig. 6b and g where we found a Ru/Al ratio of 0.05 (Tables S4 and S5, ESI†). The partial and low consumption of Al–H sites might be due to steric hindrance induced by the immobilization of the bulky Ru complex (*d*_HG-II_ ∼ 15 Å). For **C3** (*d*_pore_ = 4 nm), the amount of Ru was quantified to be 0.8 wt% corresponding to a consumption of only 7% of the available Al–H (ESI†), suggesting that Al–H sites are less accessible on this support. Investigating the Ru, Cl and N content relative to each other, we found that the Cl/Ru and N/Ru ratios remain at 2 as in the case of **HG-II**. These results, together with FT-IR results (no HCl detected),^38^ further prove that no HCl_g_ nor HCl_ads_ was released during the grafting, suggesting that the structure of the catalysts with their two chlorines is maintained.

**Fig. 4:**
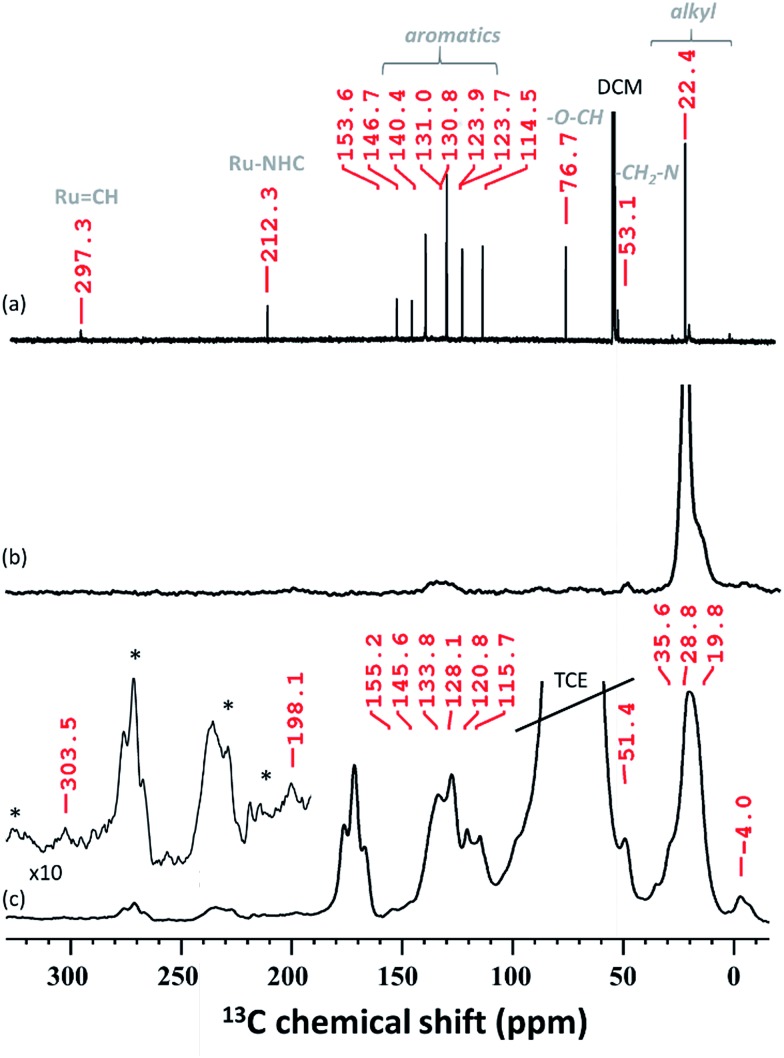
(a) ^13^C NMR (RT, 400 MHz, CD_2_Cl_2_) of **HG-II**, (b) ^13^C CP NMR (RT, 400 MHz, CD_2_Cl_2_) of **A3** and (c) ^13^C CP MAS DNP SENS spectra (100 K, 400 MHz/263 GHz) of **A3** in a 16 mM TEKPol solution in TCE. The recycle delay was 3 s, the contact time was 3 ms and the MAS frequency was 10 kHz. All characteristic resonances were obtained after 50 000 scans. The stars indicate the spinning side bands.

In solid-state NMR (SS NMR) spectroscopy, ^1^H and ^13^C signals indicate that the main functionalities of **A3** (Fig. 4b and S6, ESI†) have been incorporated into the material. Signals at around 7 ppm (^1^H) and 135 ppm (^13^C) provide evidence that aromatic functionalities are still present. Furthermore, signals at 0.9 ppm (^1^H) and 23 ppm (^13^C) are detected and mainly attributed to the alkyl residues on the surface. To improve on the sensitivity levels of conventional SS NMR, dynamic nuclear polarization surface enhanced NMR spectroscopy (DNP SENS)^39^,^40^ of **A3** has been performed. After establishing contact between **A3** and TEKPol (16 mM TEKPol in tetrachloroethane (TCE)), we were able to detect a high ^1^H solvent enhancement (*ε*_H_, defines the gain in intensity when comparing the solvent signal intensities of the microwave on/off spectra, ESI†) of 104, indicating that the radical was not destroyed as previously reported.^41^ DNP SENS analysis suggests that the carbene remains intact: a ^13^C signal at 303.5 ppm was obtained after 50 000 scans. Furthermore, a signal was found at around 198 ppm, which was assigned to Ru–C_NHC_.

**Scheme 2:**
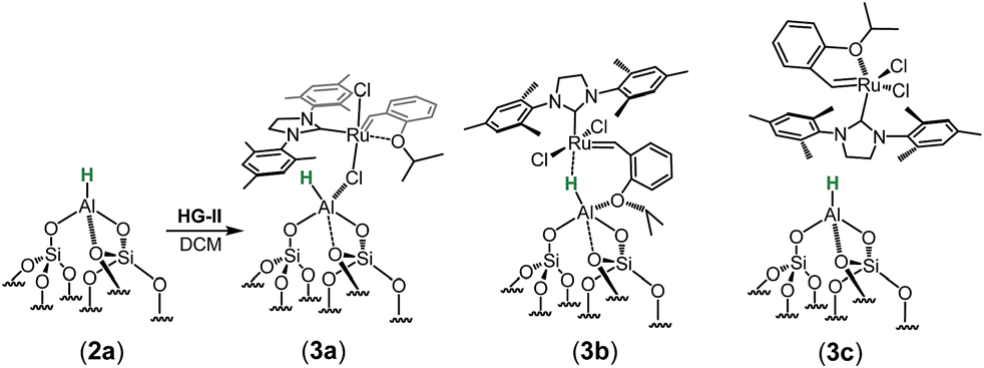
Potential interaction of **H-G-II** with **2a** leading to **3a**, **3b** or **3c**.

The spectrum obtained for the homogeneous analogue **HG-II** (Fig. 4a) shows a resonance for Ru=CH at 297 ppm and for the Ru–C_NHC_ signal at 211 ppm. Furthermore, the aromatic signals, as well as the alkyl signals, are in accordance with those of **HG-II**. To figure out which functional group of **HG-II** (iso-propoxyl, chloride or tertiary amine ligands) preferentially interacts with the [Al–H] surface groups, we performed DFT calculations (Scheme 2) assuming 3 possibilities, **3a**, **3b** and **3c**.

**Scheme 3:**
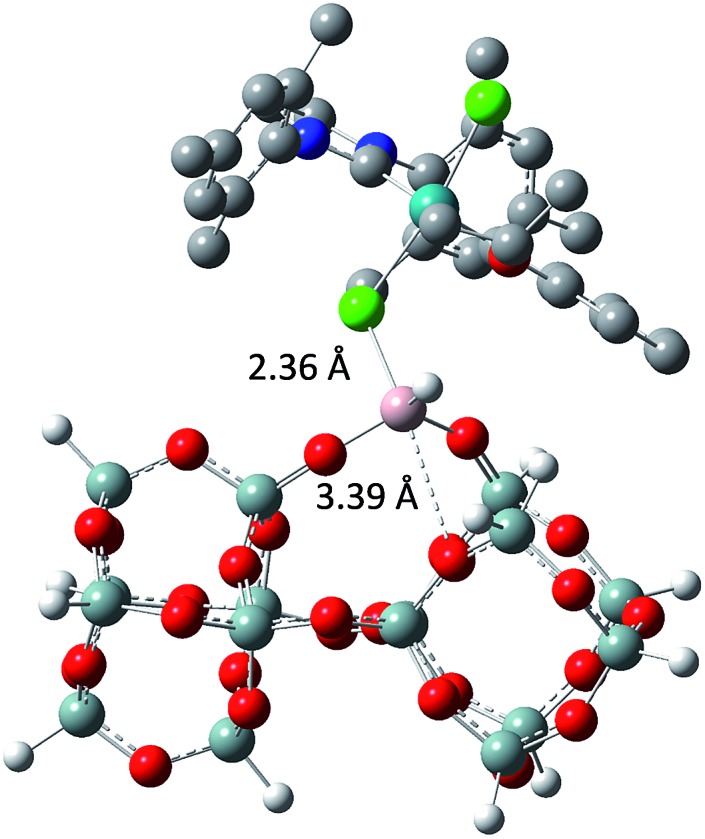
Geometry of the molecular model of **3a** using M06/TZVP//BP86/SVP (pcm = DCM).

The tertiary amine in the N-heterocyclic carbene (NHC) ligand is not accessible for reaction with Al–H due to the bulkiness of the mesityl groups (**3c** in Scheme 2). The iso-propoxy-O lone pair preferentially maintains the interaction with the [Ru]-center rather than coordinating to the Al-center (**3b** in Scheme 2) by 0.7 kcal mol^–1^. Our DFT results show that only the lone pair of the chlorine is able to interact with the Al-center releasing 3.9 kcal mol^–1^ (**3c** in Scheme 2). The lone pair of the chloride coordinates to the Al-centre (Al···Cl–Ru) which leads simultaneously (similarly to the pyridine coordination) to the opening of the Al···O–Si-interaction from 2.02 Å to 3.39 Å (Scheme 3).

The catalytic performance of the three supported catalysts **A3**, **B3** and **C3** was evaluated in RCM of diethyl-diallyl malonate (DEDAM). Unfortunately, immobilized catalysts **A3**, **B3** and **C3** are not yet compatible for reactions with functionalized olefins, as diethyldiallyl malonate (DEDAM). RCM experiments of **A3** in DCM showed (ESI†) that the catalyst leaches from the support (Fig. S12, ESI†). Leaching from **A3** is reduced using toluene (low polarity) as the solvent, because the catalyst is less soluble and remains partially confined inside the channels of SBA15, as previously described.^26^,^27^,^42^

The leaching process leads to homogeneous **HG-II** which maintains its catalytic activity. Therefore, we conclude that the adsorption of **HG-II** is a reversible process, which is expected from DFT calculations.

To study the interactions of Al–H surface groups with DEDAM and the reason for the leaching, we performed DFT calculations. DFT results show that the interactions of the DEDAM carbonyl-oxygen with the Al-center are indeed thermodynamically favoured (–1.8 kcal mol^–1^) but 2.1 kcal mol^–1^ less than those with **HG-II**. However, these findings might explain a competition between both interactions leading to the leaching of [Ru] (ESI†).

**Fig. 5:**
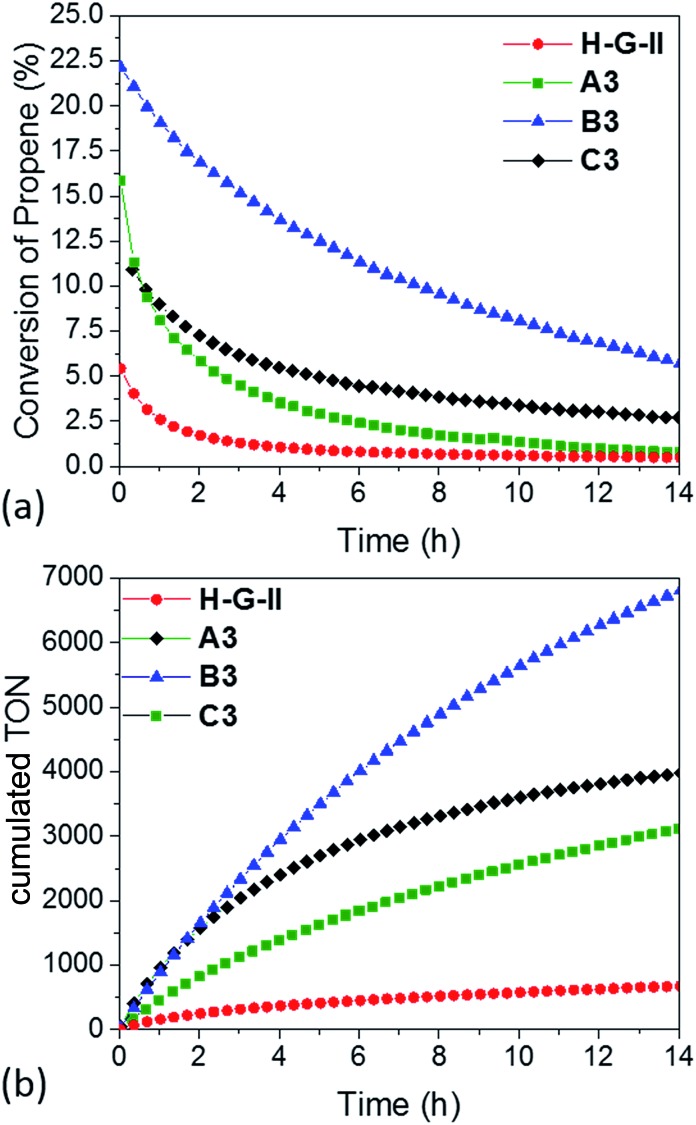
(a) Conversion and (b) cumulative TON of propene-metathesis (propene: 16 mL min^–1^; *T* = 25 °C, [Ru]: 9 μmol) over **HG-II** (red), **A3** (green), **B3** (blue) and **C3** (black).

In parallel, we investigated **A3**, **B3** and **C3**, together with the homogeneous analogue catalyst **HG-II** in propene metathesis in a continuous flow reactor (Fig. 5). The activity of immobilized catalysts **A3**, **B3** and **C3** in propene metathesis is improved in comparison to the homogeneous system by a factor of 2 (**A3**), 3 (**C3**) and even 5 for **B3**. Such an improvement was not observed by Sels *et al.*^42^ in liquid phase cyclooctene metathesis.

The maximum conversion in dynamic gas phase reaction was 5% for **HG-II**, 11% for **A3**, 15% for **C3** and 22% for **B3**. The cumulative TONs after 14 hours of reaction increase from 677 (**HG-II**) to 3113 (**A3**), 3976 (**C3**), and 6807 (**B3**). The improved activity might be explained by a decreased electron density on the metal centre due to the (Al···Cl–Ru) interaction. Electronic tuning of the anionic ligands of 2^nd^ generation Ru olefin metathesis complexes is known to change the catalytic activity of the catalyst.^9^,^43^–^45^ The activity of **B3** is enhanced compared to that of **A3** and **C3** because active sites are more accessible as they reside at the external surface of KCC-1 (**B3**).

**Fig. 6:**
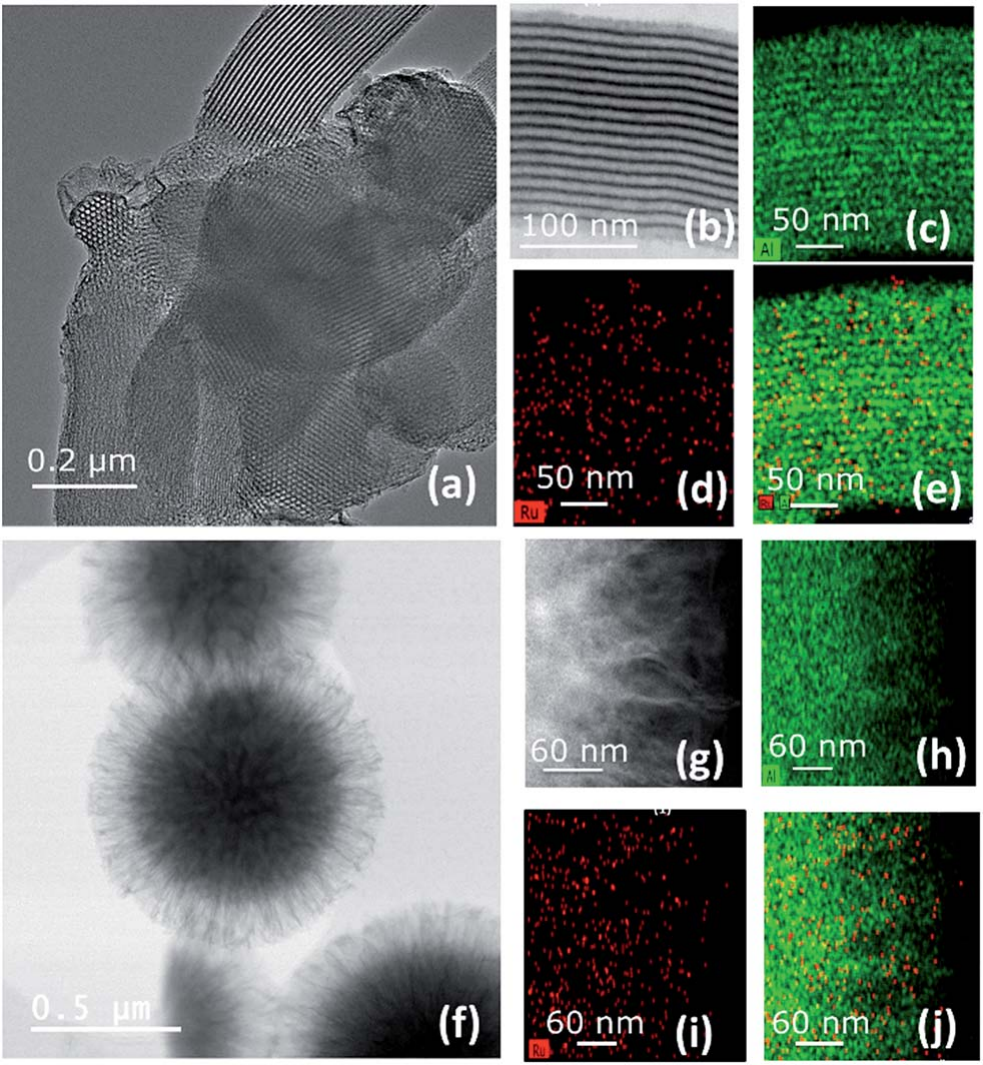
(a) Low-magnification BF-TEM analysis of **A3** (on SBA15), (b) high-magnification BF-TEM, and (c) Al (green), (d) Ru (red), and (e) Al (green) & Ru (red) superimposed elemental maps. (f) Low-magnification BF-TEM analysis of **B3** (on KCC-1), (g) high-magnification BF-TEM analysis, and (h) Al (green), (i) Ru (red), and (j) Al (green) and Ru (red) superimposed elemental maps.

To gain better understanding of the distribution of **HG-II** and its accessibility inside the mesopores of SBA15 and KCC-1 of **A3** and **B3**, we performed transmission electron microscopy (TEM) analyses (Fig. 6). Moreover, a double-aberration corrected TEM of model Titan ThemisZ from Thermo Fisher Scientific was employed to complete the mentioned analysis. It has to be noted that for each mesoporous sample, a dry specimen preparation was adapted whereby a modicum of specimen was placed onto holey-carbon coated copper grids. Spherical aberration-corrected bright-field TEM (BF-TEM) images of several particles were acquired and revealed that the structure of both materials was maintained (Fig. 6a and f). Elemental distributions of Ru and Al in both samples were determined by using the STEM-EDS spectrum imaging technique. These elemental maps contain a high degree of confidence in regard to revealing the presence of Ru and Al as these are generated by acquiring the EDS signal with a high solid-angle EDS detector of model SuperX. The elemental maps of Al are shown in Fig. 6c and h, and those of Ru in Fig. 6d and i. The superimposed Al and Ru maps are shown in Fig. 6e and j. More detailed information about EDS experiments can be found in the ESI.†

Two main pieces of information are obtained from the EF-TEM image comparing **A3** (SBA15, *d*_pore_ = 6 nm, Fig. 6a–e) and **B3** (KCC-1, *d*_pore_ = 8 nm, Fig. 6f–j). The first one is that [Ru] (red) is well-distributed on both mesoporous materials (Al = green) (Fig. 6e and j). The second one highlights a partial obstruction at the pore openings leading to the low loading of active sites at the center of the hexagonal mesopores. This explains the poor catalytic results obtained in propene metathesis, which are better using KCC-1 as a support as the active sites are more accessible.

## 3 Conclusions

The aim of this work was to investigate the type of interaction occurring between the 2^nd^ generation Hoveyda–Grubbs catalyst **HG-II** and two types of Al-modified mesoporous silica, SBA15 and KCC-1, synthesized through the SOMC concept, strategy and methodology. These mesoporous supports feature well-defined tetrahedral aluminum hydride sites having a strong Lewis acid character, [(≡Si–O–Si≡)(≡Si–O–)_2_Al–H]. Therefore, the immobilization of **HG-II** occurs through Lewis acid–base interactions as evidenced by gas phase analysis, FTIR, elemental analysis, SS NMR, DNP SENS and DFT calculations. The catalytic activity of all the materials was tested in propene metathesis. An increased activity of immobilized catalysts **A3**, **B3** and **C3** compared to their **HG-II** analogue arises from the Al···Cl–Ru interaction, making the [Ru]-center more electropositive and hence more reactive. Also the type of support affects the catalytic results. While **A3** (SBA15) is beneficial for trapping and protecting the catalyst inside the mesopores (DNP SENS, leaching), the accessibility of the active sites is reduced (lower TONs). In contrast, **B3** and **C3** (KCC-1) are more active in propene metathesis, because the active sites reside on the external surface and are hence fully accessible to the substrate.

## Conflicts of interest

There are no conflicts to declare.

## Supplementary Material

Supplementary information
