# Supplementary material for: Exploiting the interactions between the ruthenium Hoveyda–Grubbs catalyst and Al-modified mesoporous silica: the case of SBA15 vs. KCC-1
Source: Chem Sci. 2018 Mar 5;9(14):3531–7. doi: 10.1039/c7sc05200f (PMC5934738; doi:10.1039/c7sc05200f)
Supplement: Supplementary file 1 [file SC-009-C7SC05200F-s001.pdf]

Electronic Supplementary Material (ESI) for Chemical Science.
This journal is © The Royal Society of Chemistry 2018

# Electronic Supplementary Information

## Exploiting the Interactions between Ruthenium Hoveyda-Grubbs Catalyst and Al-modified Mesoporous Silica. The Case of SBA15 vs. KCC-1.

Baraa Werghi†,[a] Eva Pump†,[a] Mykyta Tretiakov,[a] Edy Abou-Hamad,[b] Andrei Gurinov, [b] Pradeep Doggali,[a] Dalaver Anjum,[b] Luigi Cavallo,[a] Anissa Bendjeriou-Sedjerari,*[a] and Jean-Marie Basset.*[a]

[a] KAUST Catalysis Center (KCC), King Abdullah University of Science and Technology, Thuwal,23955-6900, Saudi Arabia,

[b] Imaging and Characterization Lab, King Abdullah University of Science and Technology (KAUST), Thuwal, 23955-6900, Saudi Arabia

† The authors contributed equally

Jeanmarie.basset@kaust.edu.sa

Anissa.BendjeriouSedjerari@kaust.edu.sa

## Table of contents

**1. General Procedure** ........ 2

**2. Support Preparation** ........ 2

**3. Catalyst Preparation** ........ 2

**4. Gas Phase Analysis** ........ 3

**5. DFT calculations** ........ 3

**6. Infrared spectra** ........ 3

**7. Elemental analyses** ........ 8

**8. Solid-State NMR (SS NMR)** ........ 8

**9. Dynamic Nuclear Polarization Surface Enhanced NMR spectroscopy (DNP SENS)** ........ 9

**10. Nitrogen adsorption/desorption measurements** ........ 10

**11. Transmission-electron microscopy (TEM).** ........ 12

**12. Catalytic Tests** ........ 13

**13. References** ........ 14

**14. XYZ-Coordinates** ........ 15

## 1. General Procedure

All experiments were carried out under controlled atmosphere. Treatments of the surface species were carried out using high vacuum lines ($10^{-5}$ mbar) and glove box techniques. SBA15 was prepared according to literature.[1] Diisobutylaluminum hydride (DIBAL), diethyldiallyl-malonate (DEDAM), 2nd generation Hoveyda-Grubbs catalyst was purchased from Sigma Aldrich. Dichloromethane (DCM), DEDAM were dried over activated molecular sieve and degassed through freeze pump thaw cycles.

TEKPol [2] was dried under high vacuum ($10^{-5}$ mbar) and the solvents were stirred over calcium hydride and then distilled in vacuum.

## 2. Support Preparation

The reaction of mesoporous silica (dehydroxylated at 700 ºC at $10^{-5}$ mbar for 30 h) with DIBAL was carried out in a double Schlenk tube. 0.5 g of SBA-$15_{700}$ or KCC-$1_{700}$, was reacted with 1 equivalent of DIBAL (1M in hexane) followed by addition of 3 ml of dry *n*-pentane. The evolved gases were collected in a 10 L flask, and the white powder was washed with dry pentane (*2) to eliminate unreacted DIBAL. The solid was dried under vacuum ($10^{-5}$ mbar) for 12 h. The resulting solid was introduced in a glass reactor (275 mL) and heated up to 400°C (8°C/h) for 1h under dynamic vacuum ($10^{-5}$ mbar). The final amount of the aluminum grafted was 4 wt. % with an amount of Carbon of 3.51 wt. % (C/Al of 2).

## 3. Catalyst Preparation

In a double Schlenk, 250 mg of **A2, B2** or **C2** (1 eq., $n_{[Al]}$ = 0.37 mmol) was reacted at room temperature with 40 mg of complex **HG-II** [3] ($M_w$ = 626. 62 g.mol$^{-1}$ 0.2 eq., $n_{[HG\text{-}II]}$ = 0.065 mmol) in dry $CH_2Cl_2$ (8 mL). The reaction mixture was stirred for 3 hours. After contacting the supports **A2**, **B2** and **C2** with dissolved [Ru]-complex **HG-II**, the green color of the solution immediately vanished and the mesoporous silica turned brown. In comparison, previous attempts to graft **HG-II** on SBA$15_{700}$ using DCM revealed that the main part of **HG-II** remained dissolved in DCM (green solution). Only a small part of the catalyst stacked on the mesoporous silica support, which remained light green after washing. Using non-polar solvents (as toluene) [4] lowers the affinity of the catalysts for the solvent and improves its physisorption to silica oxide support.

After 3 hours the supernatant became transparent indicating the end of the reaction. After 5 h, the mixture was filtrated and the powder was washed (three times). Then, the brown powder was dried overnight and given to further investigations.

## 4. Gas Phase Analysis

After completion of the gas phase was investigated by gas chromatography on an Agilent 6850 gas chromatograph with split/split-less injector and FID. 10 µL were injected by the hot needle technique (thermospray) at an injector temperature of 250˚C using the split mode (split ration 50:1, 100 mL/min split flow). A HP-Al/KCl 50 m x 0.32 mm; 8.00 µm capillary column coated with a stationary phase divinylbenzene/ethylene glycol dimethacrylate was used with nitrogen as carrier gas at 18.3 Psi pressure. Each analysis was carried out with the same conditions: a flow rate of 2 mL/min, an isotherm at 80 ˚C for 12 min, then at 170˚C for 3 min. The detector sets with a data rate of 5 Hz and a minimum peak width of 0.04 min.

## 5. DFT calculations

To understand the structure of the catalyst after grafting **HG-II** on the support **1**, DFT calculations were performed with the generalized gradient approximation (GGA) functional with the Gaussian09 software using the BP86 level of theory of Becke and Perdew.[5-7] The electronic configuration of the molecular systems is described with the standard split-valence basis set with a polarization function of Ahlrichs and co-workers for H, C, N and O (SVP keyword in Gaussian).[8] For Ru, the small-core, quasi-relativistic Stuttgart/Dresden effective core potential, with an associated contracted valence basis set (standard SDD keyword in Gaussian09) was used.[9-10] The reported energies were obtained via single-point calculations on the BP86 optimized geometries using the M06 functional and triple-ζ basis set for main-group atoms (TZVP keyword in Gaussian09).[11-12] The influence of the solvent (DCM) was included in these single-point energy calculations by using the polarization continuum solvation model PCM.[12]

## 6. Infrared spectra

Spectra were acquired on a Nicolet 6700 FT-IR spectrometer equipped with a cell under controlled atmosphere.

**Synthesis of Al-modified mesoporous silica support**

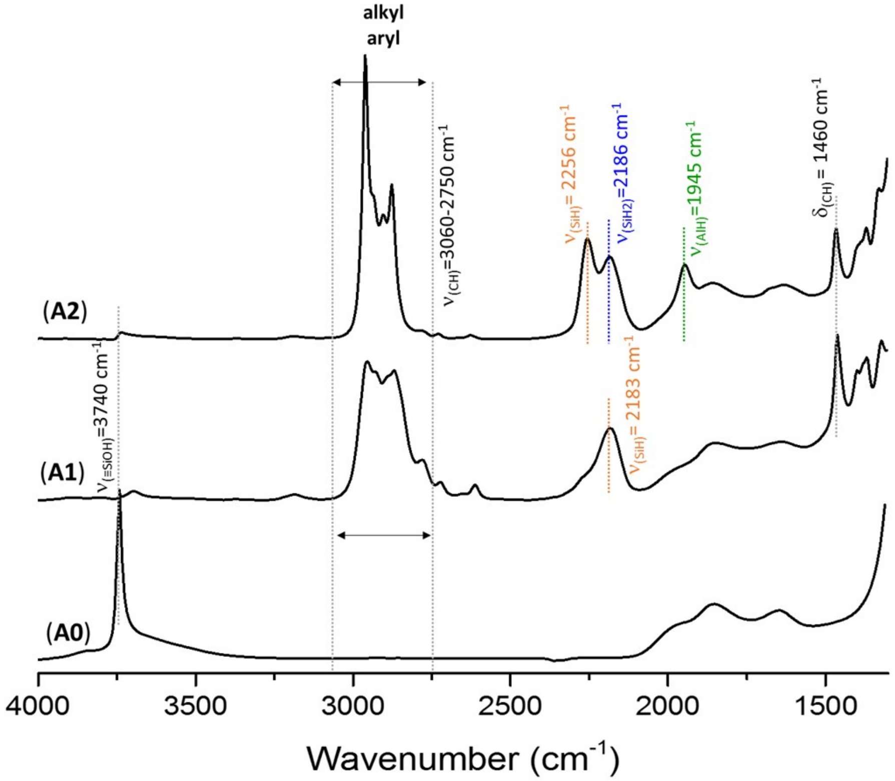


**Figure S1**. FT-IR spectra of $SBA15_{700}$ (**A0**), $Al^{i}Bu@SBA15_{700}$ (**A1**) and $Al\text{-}H@SBA15_{700}$ (**A2**)

**Pyridine adsorption**

Pyridine Transmission IR spectra were obtained by use of a Nicolet FT-IR 6700 spectrometer equipped with an DTGS -KBr detector at a 4 $cm^{-1}$ resolution with the help of specially designed Pyrex made IR cell capable for heating and evacuation; which is similar to IR cell mentioned by E. Parry.[13]

32 scans were collected to obtain each spectrum. The spectra were obtained in frequency range 400-4000 $cm^{-1}$. For FT-IR measurement, the sample in the form of a thin self-supporting wafer with a thickness less than 1 mm was mounted on pyrex cell holder. This support sample can slide back and front into the cell at proper position in front of $CaF_2$ windows to record the spectra. Thin wafers of materials, **A2, B2 and C2** supports (10 mg) were prepared by using a Carver hydraulic pellet, making unit located within glovebox under argon atmosphere with moisture level below 0.1 ppm. For wafer formation, a pressure of approximately 12,000 psi was applied. Initially, an FT-IR spectrum of clean dehydrated support was measured before pyridine adsorption treatment at room temperature for each sample.

Initially, Pyridine AR (≥99.5) (Sigma Aldrich) were purified, dried and degased using freeze- thaw pump degassing process. Then pyridine was passed over support wafer under static vacuum and allowed to adsorb. The physisorbed pyridine was removed by heating support wafer at 110°C about 1 hour and then spectra were recorded on FT-IR instrument to check pyridine adsorption. Later, spectra for given wafer recorded after heating wafer under vacuum ($10^{-6}$ mbar) bar for 1h at 200, 300 and 400 °C.

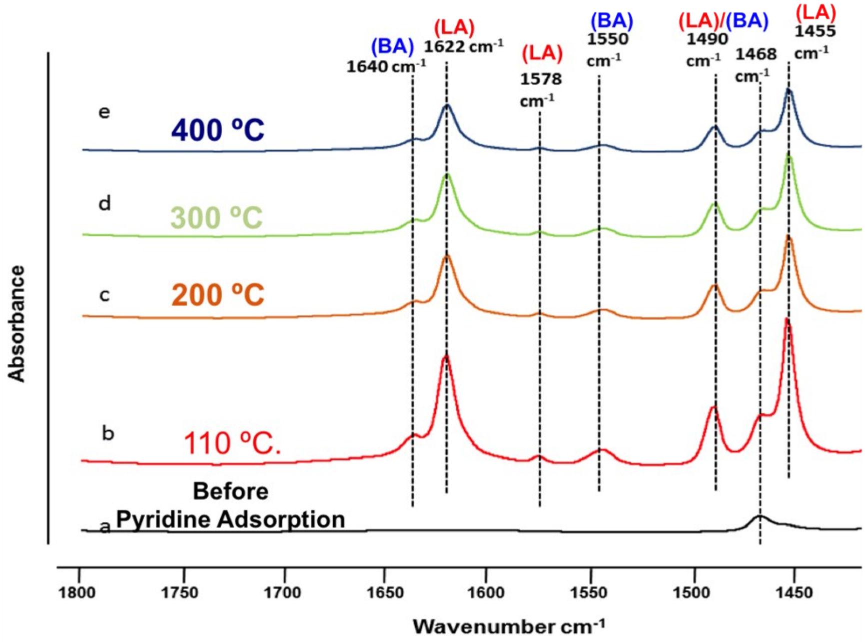


**Figure S2.** FT-IR spectra of (a) starting materials **A2**; (b) **A2** after introduction of pyridine followed by its evacuation of at 110°C; (c) at 200 °C, (d) at 300 °C (e) at 400 °C. Note: BA = Brønsted acid and LA = Lewis acid

The quantification of Lewis and Brønsted acid sites was determined as described previously [14] [15]

$$q(B,L) = \frac{A(B,L) \times \pi \times r^2}{\omega \times \varepsilon(B,L)}$$

$$\left\{ \begin{array}{c} B{:}Bronsted\ Site, \\ L{:}Lewis\ Site \\ A(B,L){:}Area\ under\ the\ peak\ 1455\ cm-1\ and\ 1550\ for\ lewis\ and\ Bronsted\ acid\ sites\ \ respectively \\ q(B,L){:}quantity\ of\ the\ correspondingacid\ sites \\ \varepsilon(B){:}Molar\ absorptivity\ coefficient\ 1.65_{cm/\ \mu mol} \\ \varepsilon(L){:}Molar\ absorptivity\ coefficient\ 2.22_{cm/\mu mol} \\ r{:}the\ radium\ of\ the\ wafer\ used\ for\ measurement,0.7\ cm \\ \omega{:}weight\ of\ the\ dry\ sample\ 10mg \end{array} \right.$$

The peaks areas at 1455 and 1550 $cm^{-1}$ were used after linearization and deconvolutions.

**Table S1.** Quantification of the Lewis and Brønsted acid sites on **A2**.

| Temperature / ˚C | Brønsted acidity (BA) / $\mu mol_{py}/g_{support}$ | Lewis acidity (LA) / $\mu mol_{py}/g_{support}$ | Total (BA+LA) / $\mu mol_{py}/g_{support}$ |
|---|---|---|---|
| **200** | 81.31 | 381.15 | 462.46 |
| **300** | 52.04 | 363.27 | 415.31 |
| **400** | 17.15 | 345.13 | 362.28 |

## Immobilization of organometallic complex HG-II

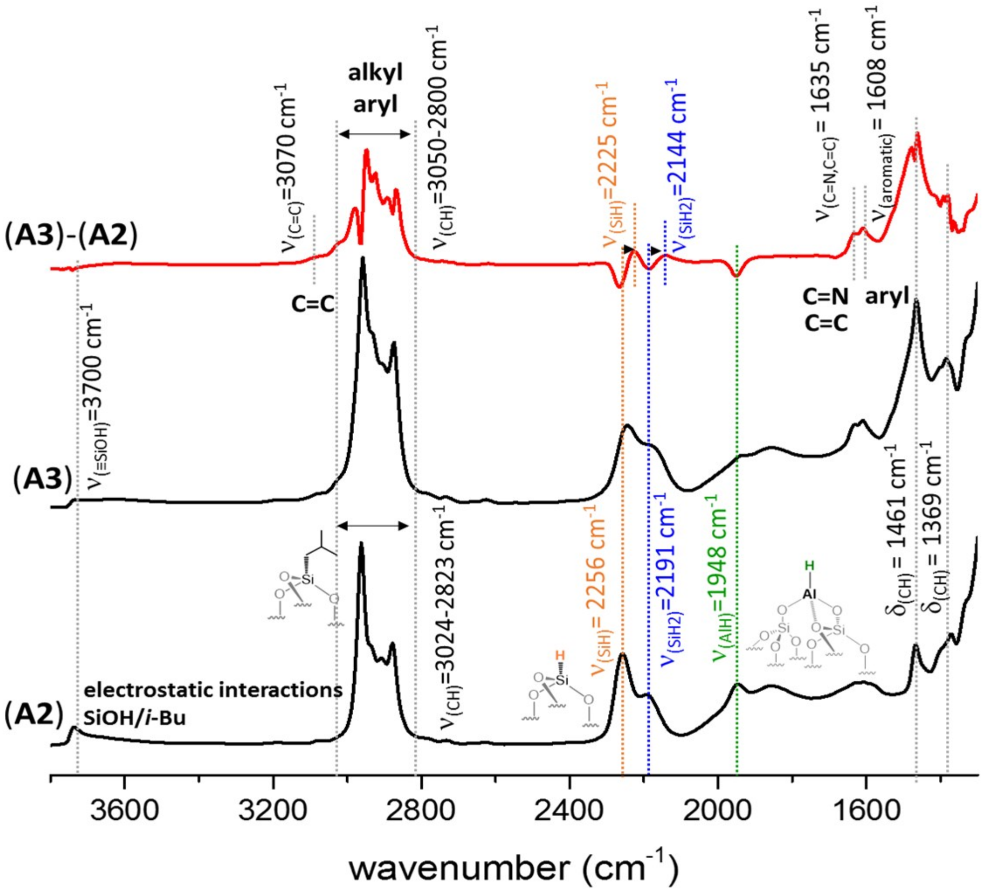


**Figure S3.** FT-IR spectra of material (**A2**), after grafting of **HG-II** (**A3**) and the subtraction (**A3**) – (**A2**) (red) in the 3900–1400 $cm^{-1}$ region at 25 °C.

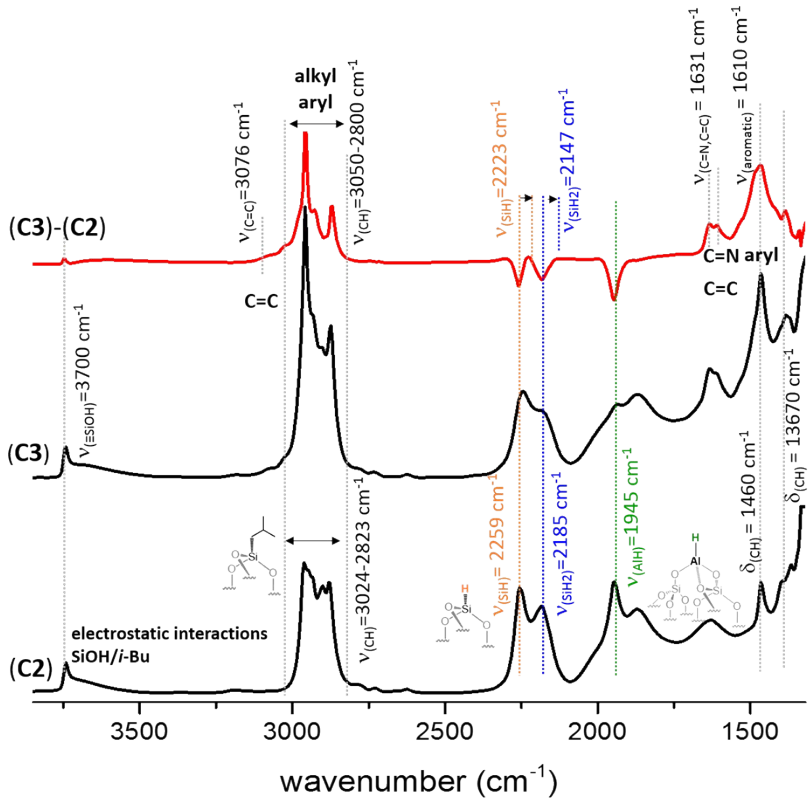


**Figure S4.** FT-IR spectra of material (**C2**), after grafting of **HG-II** (**C3**) and the subtraction (**C3**) – (**C2**) (red) in the 3900–1400 cm$^{-1}$ region at 25 °C.

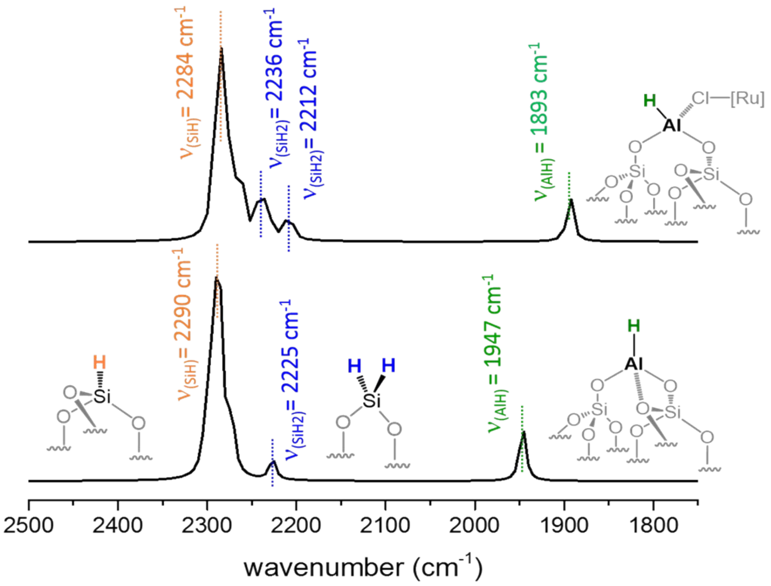


**Figure S5.** Simulated IR spectra of support (**2**), after grafting of **HG-II** (**3**) in the 3900–1400 cm$^{-1}$ region at 25 °C.

## 7. Elemental analyses

Elemental analyses (Ru, Cl, N, C) were performed at Mikroanalytisches Labor Pascher (Germany) and Varian 720-ES ICP-Optical Emission Spectrometer (Ru) after the samples preparation by microwave digestions on and on Milestone ETHOS 1. Analysis of C and N were done on Flash 2000 Elemental Analyzer from Thermo Scientific.

**Table S2**. Analytical data of materials **A2**, **B2**, **C2**, **A3**, **B3** and **C3**.

| **material** | **Ru wt.%** | **Cl wt.%** | **N wt.%** | **C wt.%** | **Cl/Ru (th.)** | **N/Ru (th.)** |
|---|---|---|---|---|---|---|
| **A2** | - | - | - | 3.5 | - | - |
| **A3** | 1.5 | 1.0 | 0.4 | 11.1 | 1.9 (2.0) | 1.9 (2.0) |
| **B2** | - | - | - | 5.3 | - | - |
| **B3** | 1.5 | - | 0.4 | 10.8 | - | 1.9 (2.0) |
| **C2** | - | - | - | 4.2 | - | - |
| **C3** | 0.8 | - | 0.2 | 7.2 | - | 2.0 (2.0) |

## 8. Solid-State NMR (SS NMR)

One-dimensional $^{1}H$ MAS and $^{13}C$ CP MAS solid-state NMR experiments were acquired on a Bruker AVANCE III spectrometer operating at 600 MHz resonance frequencies for $^{1}H$ employing a conventional double-resonance 3.2 mm probe. In all cases, the samples were packed into zirconium rotors under an inert atmosphere inside a glovebox. Dry nitrogen gas was used for sample spinning to prevent degradation of the samples. NMR chemical shifts are reported with respect to the external references TMS and adamantane. For $^{13}C$ CP MAS NMR experiments, the following parameters were used: a 90° proton pulse length of 2.9 µs, a contact time of 5 ms, with high-power proton decoupling a recycle delay of 5 s and the number of scans was 18 000.

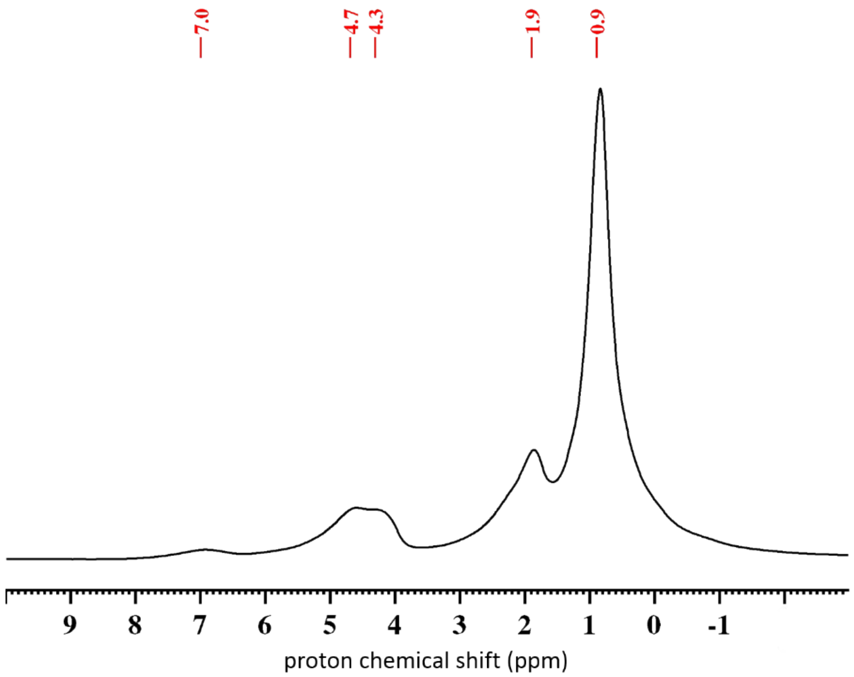


**Figure S6**. One dimensional (1D) $^{1}H$ MAS NMR spectrum of **A3** acquired at room temperature with 10 kHz MAS and 18 000 scans.

## 9. Dynamic Nuclear Polarization Surface Enhanced NMR spectroscopy (DNP SENS)

DNP enhanced solid-state NMR experiments were conducted on a 400 MHz Bruker Avance III solid-state NMR spectrometer using a 3.2 mm Bruker triple resonance low-temperature magic angle spinning (LTMAS) probe. The experiments were performed at ca. 100 K with a 263 GHz gyrotron. The sweep coil of the main magnetic field was set for the microwave irradiation occurring at the $^{1}H$ positive enhancement maximum of the TEKPol biradical.

*Sample Preparation.* 20 mg of finely ground catalyst **A3** ($c_{[Ru]}$ = 2.3 µmol) was impregnated with 40 µL of a biradical solution consisting of 16 mM TEKPol in 1,1,2,2-tetrachloroethane (TCE) ($c_{TEKPol}$ = 0.6 $µmol_{TEKPol}$/sample corresponding to a radical concentration $c_{radical}$ = 1.2 $µmol_{radical}$/sample). Hence, the [Ru]/[radical]-ratio is ≥ 1, meaning that a complete destruction of the radical can be excluded, because no solvent $^{1}H$ enhancement should be obtained. In a typical experiment, the sample was packed into a sapphire rotor capped with a Teflon plug under argon atmosphere and then immediately inserted into the pre-cooled DNP probe for experiments.

The probe configuration used triple mode for $^{1}H$ and $^{13}C$. The amplitude of $^{1}H$ spin lock was ramp 100.50.100 to have the maximum value. DNP enhancement factors (ε) were measured by integrating the intensity of spectra acquired with and without continuous wave irradiation. For $^{13}C$ NMR experiments, the acquisition parameters used are 3 s repetition delays, a $^{1}H$ π/2 pulse length of 2.5 µs to afford 100 kHz $^{1}H$ decoupling using the SPINAL 64 decoupling method.

A 2 ms contact time was used for cross polarization experiments. The MAS frequency was 10 kHz. All $^{13}C$ NMR spectra were referenced to adamantane with the higher frequency peak set to 38.48 ppm with respect to TMS (0 ppm).

## 10. Nitrogen adsorption/desorption measurements

Typical isotherms of all materials are shown in Figure S6-8. It is a classical type IV isotherm, which bears a characteristic H1 hysteresis loop. A type IV isotherm indicates the monolayer adsorption followed by multilayer formation and capillary condensation.[16]

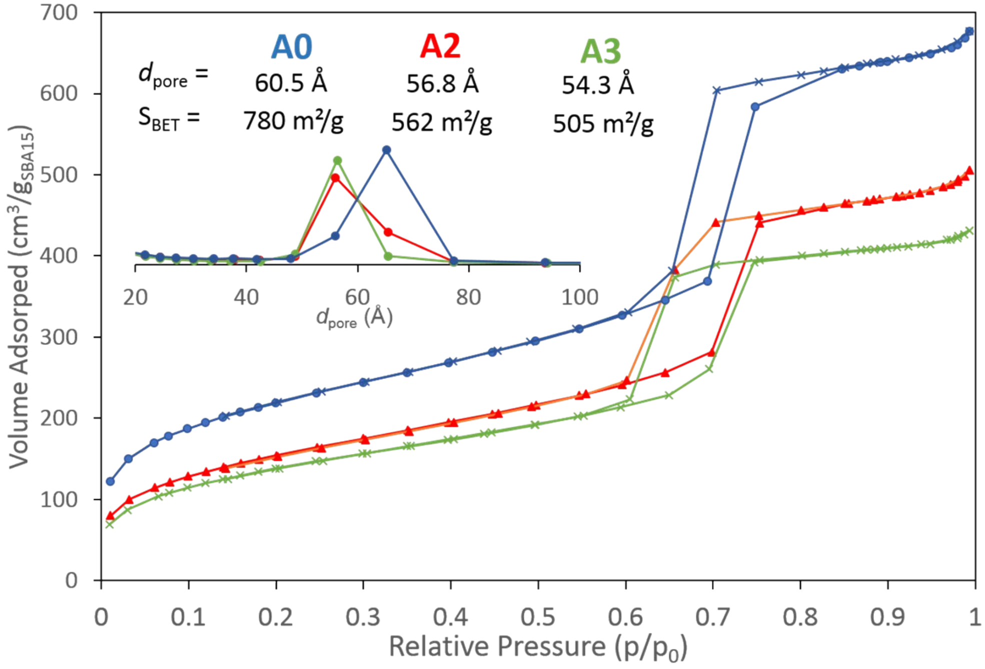


**Figure S7.** Nitrogen adsorption/desorption isotherms at 77 K of **A0** (blue), **A2** (red) and **A3** (green).

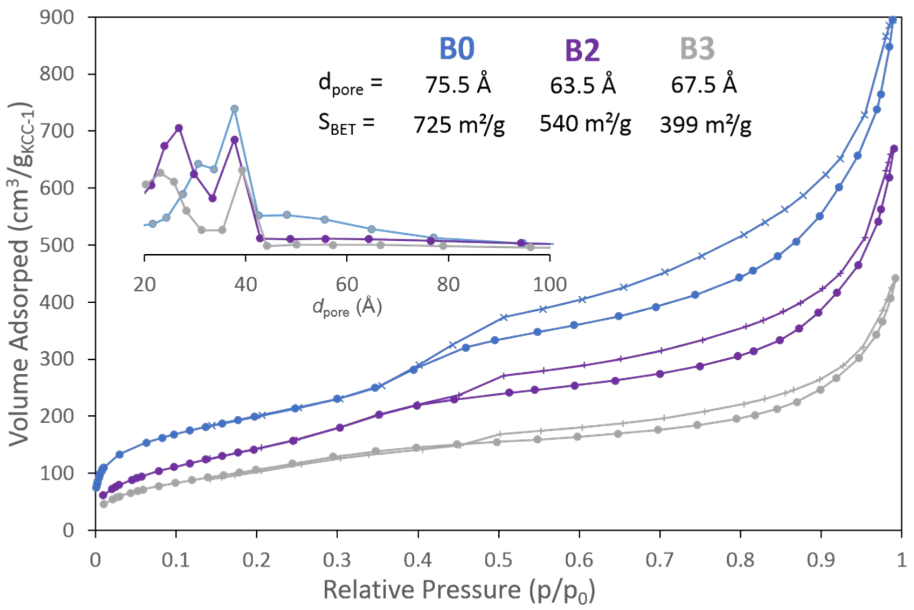


**Figure S8.** Nitrogen adsorption/desorption isotherms at 77 K of **B0** (blue), **B2** (violet) and **B3** (grey).

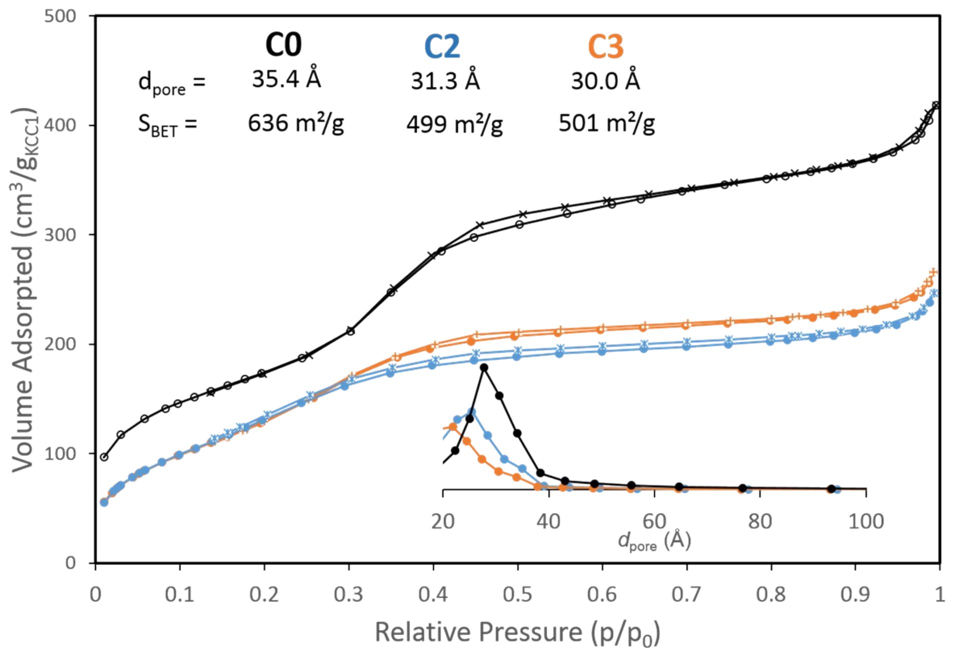


**Figure S9.** Nitrogen adsorption/desorption isotherms at 77 K of **C0** (black), **C2** (blue) and **C3** (orange).

## 11. Transmission-electron microscopy (TEM).

The primary particle size and morphology of **A3** and **B3** were examined by transmission electron microscopy (TEM). This task was accomplished by using a double aberration corrected (double Cs-corrected) microscope of model Titan 80-300 ThemisZ from Thermo-Fisher Scientific (Waltham, MA). Samples were loaded onto holey-carbon coated copper grids for TEM analysis. The samples were placed onto grids in a solvent-free but ambient environment. Conventional bright-field TEM (BF-TEM) imaging-technique was performed by operating the scope at the accelerating voltage of 300 kV. Furthermore, the microscope was set to a range of magnifications during the image acquisition process. No nanoparticles of Ru were obtained, proving that Ru was not decomposed during the grafting. It is pertinent to note that the X-ray energy dispersive (EDS) analysis of samples was also done for the determining the overall elemental composition. Scanning TEM (STEM) analysis in conjunction with EDS was performed to determine the spatial distributions of elements in the samples. The focus of STEM-EDS analysis was to generate the elemental maps of Al and Ru elements only. While the mapping of Si and O were not considered because the atomic structure of SBA15 is well documented in literature.26 Therefore, it was sufficient to detect the distribution of Al for revealing its complete presence on SBA15 regions in the generated maps. To perform the elemental mapping analysis, we selected Al-K peak (1.51 keV) and Ru-L Peak (2.56 keV) for Al and Ru elements, respectively. The STEM or TEM images were acquired with Gatan Microscopy Suite of version GMS 2.3 from Gatan while the STEM-EDS spectrum-image datasets are acquired and processed in Esprit Software from Bruker, Inc.

Structures of **A3** and **B3** were found to be well preserved after their loading with **HG-II** as presented in the main paper (Figure 6). For **A3**, both channels and walls of the silica particles could be identified easily. The presence of Ru in the channels was proved by the Ru-maps and Al-maps. The measurements on the white dot matched with channel width and hence it corroborates our argument that the Ru was present inside the channels. For **B3**, the Ru maps and Al-maps proved the presence of Ru throughout the external surface.

EDS spectra for **A3** and **B3** can be found in Figure S10 and S11. Both spectra confirm the presence of Ru, Cl, Al, Si, O, C (for edges see Table S3), which are the main components of the catalyst **A3** and **B3**.

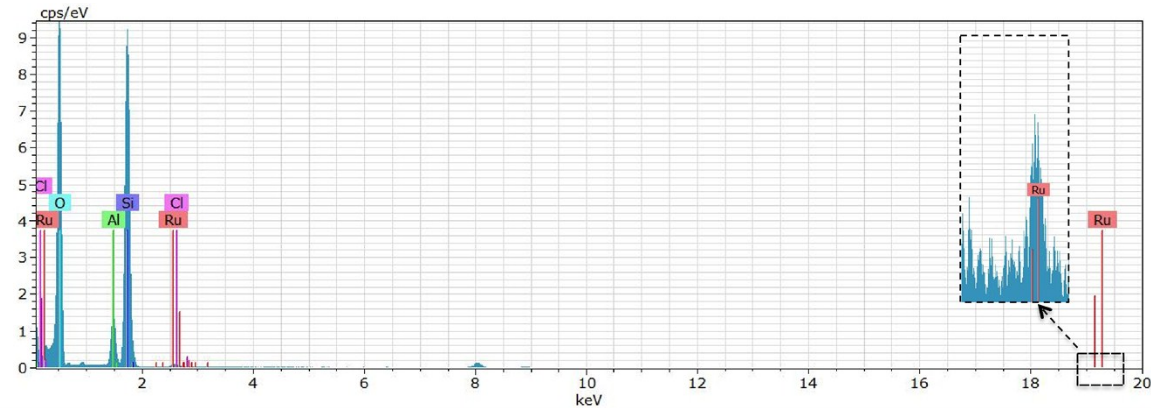


**Figure S10**. Energy dispersed spectrum (EDS) for **A3.**

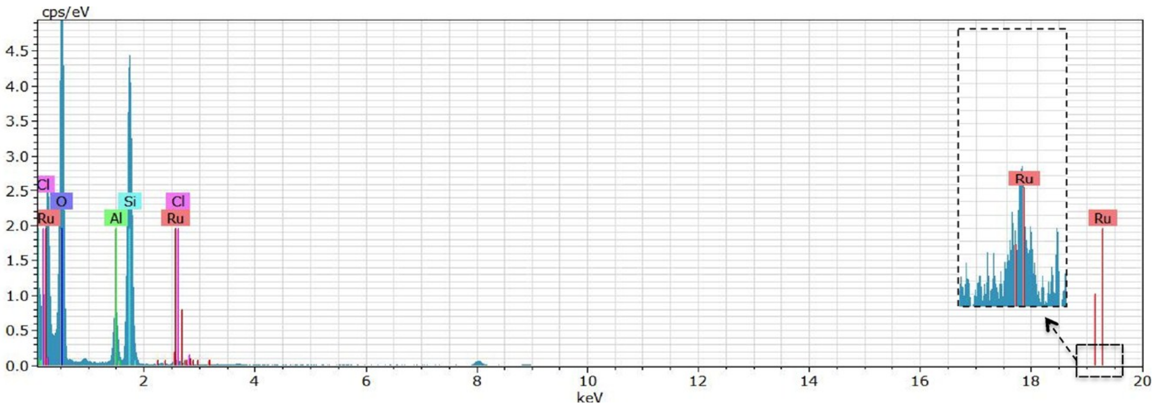


**Figure S11.** Energy dispersed spectrum (EDS) for **B3.**

**Table S3**. EDS edges (in keV) of elements under investigation.

| Energy (keV) | Ru | Al | Cl | O | Si |
|---|---|---|---|---|---|
| **Kα** | 19.233 | 1.486 | 2.621 | 0.525 | 1.739 |
| **Lα** | 2.558 | - | - | - | - |

The low ruthenium (Ru) signal on the elemental map indicates that only a small fraction of Ru is presented in both samples, **A3** and **B3**. By element quantification, we found a Ru/Al ration of 0.05 (Table S4 and Table S5).

**Table S4**. Elements quantification of **A3** by EDS spectrum analysis.

| **Elements** | **AN** | **Series** | **Net** | **Mass C. [Wt.%]** | **Norm. C. [W.%]** | **Atom C. [at.%]** | **Error (Wt.%]** |
|---|---|---|---|---|---|---|---|

| | | | | | | | |
|---|---|---|---|---|---|---|---|
| **O** | 8 | K series | 49548 | 26184.63 | 48.73 | 62.87 | 0.11 |
| **Si** | 14 | K series | 61346 | 22354.4 | 41.6 | 30.57 | 0.059 |
| **Al** | 13 | K series | 8936 | 4394.04 | 8.18 | 6.26 | 0.07 |
| **Ru** | 44 | L series | 1475 | 799.43 | 1.49 | 0.3 | 0.05 |
| | | | Total | 53732.49 | 100 | 100 | |

**Table S5.** Elements quantification of **B3** by EDS spectrum analysis.

| **Elements** | **AN** | **Series** | **Net** | **Mass C. [Wt.%]** | **Norm. C. [Wt.%]** | **Atom C. [at.%]** | **Error (Wt.%)** |
|---|---|---|---|---|---|---|---|
| **O** | 8 | K series | 25722 | 37831.75 | 53.99 | 67.71 | 0.12 |
| **Si** | 14 | K series | 24576 | 25063.51 | 35.77 | 25.55 | 0.06 |
| **Al** | 13 | K series | 4369 | 6049.22 | 8.63 | 6.42 | 0.07 |
| **Ru** | 44 | L series | 935 | 1121.43 | 1.6 | 0.32 | 0.06 |
| | | | Total | 70065.91 | 100 | 100 | |

## 12. Catalytic Tests

### Propene Metathesis

Catalytic propylene metathesis was performed on a micropilot (PID Eng&Tech) equipped with a stainless steel reactor at atmospheric pressure. The catalyst powder was placed in the reactor supported by preliminary dried quartz glass wool. Conversions were calculated based on carbon mass balance.

Typical procedure: **HG-II** (57 mg, n = 0.091 mmol), **A3**, **B3** (60 mg, loading 1.5 % of Ru based on ICP-OES, 0.0089 mmol) and **C3** (120 mg, loading 0.8 % of Ru based on ICP-OES, 0.0095 mmol) was charged within a glove-box, into a 0.5 inch diameter stainless steel reactor fitted with a four-channels isolating valve. After connection to the rack, all the tubing was purged by a mixture of helium (80 mL/min) and argon (30 mL/min) flow for 30 minutes, then with argon flow (30 mL/min) for additional 30 minutes. A mixture of propylene (16 mL/min) and argon (5 mL/min) as internal standard was then sent for 5 minutes bypassing

the reactor. The four-channel valve was then switched on and the mixture was reacted at room temperature at 1 atm. The gas products were analyzed on line with a GC.

The gas products were analyzed by on-line gas chromatography using a Varian 450-GC system with a flame-ionization detector (FID) for analysis of hydrocarbons and thermal conductivity detector (TCD) for quantification of argon as internal standard. Column: GS-GASPRO; 30 m length, 0.32 mm diameter (Agilent J&W GC Columns). GC was calibrated and quantification of the products was performed using calibration mixture. Retention times for each component of the calibration mixture was established based on at least 3 independent runs and used for calibration curve. Method: Injector setpoint: 220 °C. Oven: column temperature was programmed from 40 °C (hold for 2 min) to 180 °C at 10 °C/min rate. FID 250 °C, TCD 175 °C.

**RCM Metathesis**

The activity of **A3** and **B3** were examined in ring closing metathesis of the benchmark substrate diethyldially malonate (DEDAM). The reaction was performed under inert Argon atmosphere in a Schlenk. **A3** (14 mg, 0.0021 mmol, 200 eq.) and **B3** (13 mg, 0.0018 mmol, 230 eq.) was dispersed in DCM or toluene (10 mL) under constant stirring. Then, DEDAM (100 µL, 0.416 mmol, 1 eq.) was added. The progress of the reaction was monitored by GC every couple of hours. A split test was performed for A3 in DCM under the same conditions. After 1 h the conversion of the reaction was checked by GC. The remaining reaction mixture (8 mL) was split into two halves: one part (4 mL) was continued to stirr, the other part (4 mL) was filtered under inert conditons and transferred to another dry, Ar-filled Schlenk. After 2 h, both samples were analyzed by GC. Results show (Figure S12) that the [Ru] almost fully leached from the support due to the interaction of DEDAM with the Lewis-acidic Al-sites, suggesting that the catalytic activity is mainly due to the leached catalyst.

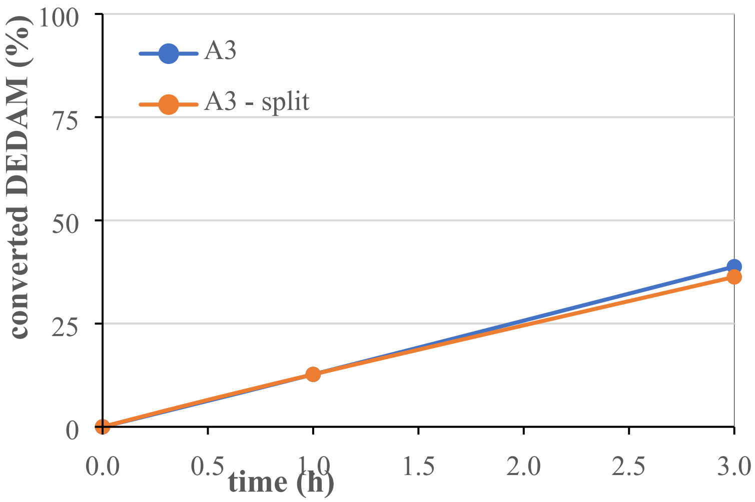


**Figure S12.** Split test of **A3** with DEDAM in DCM. At 1 h, the sample was analyzed before being split: blue curve – non-filtered sample, orange: filtered sample.

## 13. References


1. Zhao, D.; Huo, Q.; Feng, J.; Chmelka, B. F.; Stucky, G. D., Nonionic Triblock and Star Diblock Copolymer and Oligomeric Surfactant Syntheses of Highly Ordered, Hydrothermally Stable, Mesoporous Silica Structures. *Journal of the American Chemical Society* **1998,** *120* (24), 6024-6036.
2. Zagdoun, A.; Casano, G.; Ouari, O.; Schwaerzwalder, M.; Rossini, A. J.; Aussenac, F.; Yulikov, M.; Jeschke, G.; Coperet, C.; Lesage, A.; Tordo, P.; Emsley, L., Large Molecular Weight Nitroxide Biradicals Providing Efficient Dynamic Nuclear Polarization at Temperatures up to 200 K. *Journal of the American Chemical Society* **2013,** *135* (34), 12790-12797.
3. Garber, S. B.; Kingsbury, J. S.; Gray, B. L.; Hoveyda, A. H., Efficient and Recyclable Monomeric and Dendritic Ru-Based Metathesis Catalysts. *Journal of the American Chemical Society* **2000,** *122* (34), 8168-8179.
4. Van Berlo, B.; Houthoofd, K.; Sels, B. F.; Jacobs, P. A., Silica Immobilized Second Generation Hoveyda-Grubbs: A Convenient, Recyclable and Storageable Heterogeneous Solid Catalyst. *Advanced Synthesis & Catalysis* **2008,** *350* (13), 1949-1953.
5. Becke, A. D., Density-functional exchange-energy approximation with correct asymptotic behavior. *Phys. Rev. A* **1988,** *38* (6), 3098-3100.
6. Perdew, J. P., Erratum: Density-functional approximation for the correlation energy of the inhomogeneous electron gas. *Phys. Rev. B* **1986,** *34* (10), 7406-7406.
7. Perdew, J. P., Density-functional approximation for the correlation energy of the inhomogeneous electron gas. *Phys. Rev. B* **1986,** *33* (12), 8822-8824.
8. Schafer, A.; Horn, H.; Ahlrichs, R., Fully optimized contracted gaussian-basis sets for atoms li to kr. *Journal of Chemical Physics* **1992,** *97* (4), 2571-2577.

9. Haussermann, U.; Dolg, M.; Stoll, H.; Preuss, H.; Schwerdtfeger, P.; Pitzer, R. M., Accuracy of energy-adjusted quasi-relativistic abinitio pseudopotentials - all-electron and pseudopotential benchmark calculations for hg, hgh and their cations. *Molecular Physics* **1993,** *78* (5), 1211-1224.
10. Leininger, T.; Nicklass, A.; Stoll, H.; Dolg, M.; Schwerdtfeger, P., The accuracy of the pseudopotential approximation. II. A comparison of various core sizes for indium pseudopotentials in calculations for spectroscopic constants of InH, InF, and InCl. *The Journal of Chemical Physics* **1996,** *105* (3), 1052.
11. Barone, V.; Cossi, M., Quantum calculation of molecular energies and energy gradients in solution by a conductor solvent model. *J. Phys. Chem. A* **1998,** *102* (11), 1995-2001.
12. Tomasi, J.; Persico, M., Molecular-interactions in solution - an overview of methods based on continuous distributions of the solvent. *Chemical Reviews* **1994,** *94* (7), 2027-2094.
13. Parry, E. P., An infrared study of pyridine adsorbed on acidic solids. Characterization of surface acidity. *Journal of Catalysis* **1963,** *2* (5), 371-379.
14. Barzetti, T.; Selli, E.; Moscotti, D.; Forni, L., Pyridine and ammonia as probes for FTIR analysis of solid acid catalysts. *Journal of the Chemical Society, Faraday Transactions* **1996,** *92* (8), 1401-1407.
15. González-Rodríguez, B.; Trujillano, R.; Rives, V.; Vicente, M. A.; Gil, A.; Korili, S. A., Structural, textural and acidic properties of Cu-, Fe- and Cr-doped Ti-pillared montmorillonites. *Applied Clay Science* **2015,** *118*, 124-130.
16. Thommes, M.; Kaneko, K.; Neimark, A. V.; Olivier, J. P.; Rodriguez-Reinoso, F.; Rouquerol, J.; Sing, K. S. W., Physisorption of gases, with special reference to the evaluation of surface area and pore size distribution (IUPAC Technical Report). *Pure and Applied Chemistry* **2015,** *87* (9-10), 1051-1069.

## 14. XYZ-Coordinates

**Molecular Model – Surface Al-H**

| | | | |
|---|---|---|---|
| H | -1.72119900 | 4.11263300 | 1.93271200 |
| Al | -1.37100600 | 2.57946600 | 1.77806500 |
| O | -2.35281000 | 1.45823100 | 2.68886300 |
| Si | -2.92909300 | -0.03075200 | 3.01074300 |
| O | -1.90036300 | -1.21262100 | 2.39916800 |
| Si | -1.05185800 | -1.68951700 | 1.08359200 |
| O | -1.91476700 | -2.77226500 | 0.17618800 |
| Si | -3.40723000 | -2.90746900 | -0.54937300 |
| O | -4.57904300 | -2.25752900 | 0.42369900 |
| Si | -5.39984700 | -1.03427000 | 1.20156800 |
| O | -5.94550500 | 0.05343700 | 0.05936800 |
| Si | -5.63713800 | 1.01776200 | -1.24360400 |
| O | -4.87316400 | 0.15525700 | -2.45183300 |

| | | | |
|---|---|---|---|
| Si | -3.42206700 | -0.60390200 | -2.73608400 |
| O | -3.36843800 | -2.08747300 | -2.00539700 |
| O | -1.93875900 | 2.13518100 | -0.10471700 |
| Si | -1.13336600 | 0.86838600 | -0.97534400 |
| O | -0.75863600 | -0.33627200 | 0.08583900 |
| O | 0.33280200 | 2.18060700 | 1.66426000 |
| Si | 1.69148800 | 1.32717400 | 1.50769600 |
| O | 2.10522800 | 0.98581700 | -0.07321300 |
| Si | 1.75226400 | 1.10720600 | -1.66882400 |
| O | 2.98078900 | 2.08108700 | -2.22283800 |
| Si | 4.55745100 | 1.85977400 | -1.61566400 |
| O | 4.74107500 | 2.24500300 | -0.00543100 |
| Si | 4.56649100 | 1.75575900 | 1.58587000 |
| O | 4.80131700 | 0.11600400 | 1.77543700 |
| Si | 4.79338700 | -1.53478600 | 1.57794800 |
| O | 3.28595200 | -2.21820800 | 1.92178700 |
| Si | 1.83221800 | -1.67204600 | 1.32007900 |
| O | 0.36158100 | -2.43475500 | 1.55270300 |
| Si | -3.20518000 | 3.08930500 | -0.86517200 |
| O | -4.63854700 | 2.27497600 | -0.77348100 |
| O | -2.22210500 | 0.38244000 | -2.10246800 |
| O | 0.14947900 | 1.60153300 | -1.72916800 |
| O | 1.78252300 | -0.33311800 | -2.48897600 |
| Si | 2.23645500 | -1.85127100 | -1.95232300 |
| O | 3.84154800 | -2.12372000 | -2.29383300 |
| Si | 5.17109200 | -1.37861300 | -1.60167900 |
| O | 4.99738300 | 0.26420300 | -1.79464500 |
| O | 2.01814100 | -1.80820200 | -0.31753500 |
| O | 1.57885300 | -0.22223200 | 2.09698400 |
| O | 3.02174400 | 2.10996500 | 2.12621800 |
| O | 5.24777300 | -1.82029400 | 0.00048900 |
| O | -4.41520700 | -0.25711300 | 2.27042800 |

| | | | |
|---|---|---|---|
| H | -3.21605600 | -0.75556700 | -4.19126500 |
| H | 5.42923500 | 2.74006300 | -2.42396600 |
| H | 5.54884500 | 2.49826600 | 2.40644000 |
| H | 5.76284200 | -2.17228500 | 2.49494900 |
| H | -2.76390600 | 3.29881800 | -2.27421500 |
| H | -3.08702300 | -0.28149400 | 4.46285800 |
| H | -3.68853800 | -4.33390700 | -0.81446500 |
| H | -6.90534700 | 1.56829500 | -1.76803800 |
| H | -6.57993600 | -1.60651300 | 1.88728600 |
| H | -3.28805800 | 4.32770900 | -0.05290400 |
| H | 6.39520500 | -1.83851600 | -2.29399500 |
| H | 1.41533900 | -2.88848800 | -2.61542300 |

**Pyridine**

| | | | |
|---|---|---|---|
| C | -1.14903400 | -0.72881200 | 0.00000300 |
| C | -1.20723900 | 0.67712600 | 0.00000400 |
| C | -0.00013400 | 1.39563300 | -0.00001200 |
| C | 1.20712300 | 0.67731000 | -0.00000200 |
| C | 1.14918100 | -0.72859700 | 0.00001300 |
| H | -2.08244100 | -1.32246100 | 0.00001000 |
| H | -2.17967600 | 1.19449300 | 0.00002300 |
| H | -0.00018200 | 2.49760500 | 0.00000400 |
| H | 2.17943200 | 1.19491900 | 0.00001100 |
| H | 2.08265900 | -1.32212800 | 0.00001000 |
| N | 0.00011700 | -1.42834300 | -0.00001400 |

**HG-II**

| | | | |
|---|---|---|---|
| C | -0.25078300 | -1.45781600 | -0.00320900 |
| Ru | -0.14736300 | 0.50130600 | -0.04440300 |
| Cl | -0.62792000 | 0.79500000 | 2.25433500 |
| Cl | -0.54462600 | 0.64321800 | -2.36864000 |
| N | -1.46358300 | -2.12547500 | -0.00958100 |

| | | | |
|---|---|---|---|
| N | 0.72177500 | -2.43214200 | 0.00727900 |
| C | -1.31975500 | -3.58486300 | -0.15925600 |
| C | 0.17388900 | -3.79923600 | 0.11717000 |
| H | 0.66070700 | -4.47701300 | -0.61427600 |
| H | 0.36416500 | -4.21095700 | 1.13511100 |
| H | -1.60797400 | -3.89986900 | -1.18810000 |
| H | -1.97706900 | -4.12284100 | 0.55486000 |
| C | 2.14684200 | -2.28120000 | 0.06769400 |
| C | 2.88455600 | -2.28674700 | -1.14429300 |
| C | 4.28791800 | -2.18788500 | -1.06899800 |
| C | 4.96478300 | -2.09296700 | 0.16479900 |
| C | 4.19683100 | -2.08869600 | 1.34699300 |
| C | 2.79031000 | -2.18442900 | 1.32823000 |
| H | 4.86897900 | -2.17985600 | -2.00674800 |
| H | 4.70679400 | -2.00077600 | 2.32126600 |
| C | -2.78325000 | -1.54758900 | -0.02653200 |
| C | -3.46442800 | -1.36512400 | 1.21188000 |
| C | -4.75839200 | -0.81172300 | 1.18650300 |
| C | -5.40594700 | -0.47151400 | -0.01901300 |
| C | -4.74131700 | -0.75323100 | -1.22625100 |
| C | -3.44714900 | -1.31388300 | -1.26120500 |
| H | -5.28329300 | -0.65755200 | 2.14447900 |
| H | -5.25039700 | -0.54865300 | -2.18341500 |
| C | 1.67568900 | 0.79788700 | -0.04472200 |
| H | 2.44305500 | 0.00297700 | -0.00107100 |
| C | 2.18944500 | 2.15300100 | -0.13107000 |
| C | 3.57667500 | 2.44197900 | -0.19169300 |
| C | 1.26698100 | 3.24229600 | -0.16807600 |
| C | 4.03453300 | 3.76271500 | -0.29102500 |
| H | 4.28706600 | 1.60002700 | -0.16176000 |
| C | 1.71837000 | 4.56918900 | -0.26852500 |
| C | 3.10340600 | 4.81744200 | -0.33128300 |

| | | | |
|---|---|---|---|
| H | 5.11375000 | 3.97478400 | -0.33751400 |
| H | 1.01369100 | 5.41128000 | -0.29847800 |
| H | 3.45436200 | 5.85873100 | -0.41060800 |
| O | -0.04126400 | 2.84705200 | -0.10909200 |
| C | -1.12675200 | 3.83407600 | -0.00467300 |
| H | -0.88682700 | 4.63601300 | -0.73813600 |
| C | -2.41368400 | 3.14113000 | -0.43633500 |
| H | -2.30576400 | 2.69198600 | -1.44263800 |
| H | -2.69309700 | 2.34261700 | 0.28156100 |
| H | -3.23505500 | 3.88646400 | -0.45906100 |
| C | -1.18944300 | 4.39440300 | 1.41658700 |
| H | -1.99474900 | 5.15507000 | 1.48302800 |
| H | -1.40328100 | 3.57443300 | 2.13092900 |
| H | -0.23760100 | 4.87579000 | 1.71717900 |
| C | -2.84727500 | -1.79240400 | 2.52131900 |
| H | -3.58722800 | -1.72713800 | 3.34317900 |
| H | -2.47889400 | -2.83951200 | 2.47728400 |
| H | -1.97986700 | -1.14738700 | 2.77831400 |
| C | -2.86312600 | -1.74049500 | -2.58760000 |
| H | -3.21372500 | -2.76559800 | -2.84586300 |
| H | -3.19384300 | -1.06775000 | -3.40312900 |
| H | -1.75849900 | -1.73088200 | -2.58298800 |
| C | -6.77675900 | 0.16720500 | -0.00843100 |
| H | -6.70545400 | 1.25868300 | 0.19396200 |
| H | -7.29412100 | 0.04651700 | -0.98147600 |
| H | -7.42325300 | -0.26483000 | 0.78328800 |
| C | 1.99428700 | -2.12478200 | 2.61116300 |
| H | 1.31436100 | -1.24483500 | 2.62516900 |
| H | 1.35464900 | -3.02315100 | 2.74704100 |
| H | 2.66522100 | -2.05817800 | 3.48980200 |
| C | 2.17661500 | -2.34352900 | -2.47759800 |
| H | 1.56128800 | -3.26332000 | -2.57991600 |

| | | | |
|---|---|---|---|
| H | 1.48408200 | -1.48254000 | -2.60064800 |
| H | 2.90221900 | -2.33055200 | -3.31426200 |
| C | 6.47543400 | -2.02626700 | 0.21727800 |
| H | 6.92029800 | -3.04594600 | 0.19988400 |
| H | 6.89202900 | -1.47759200 | -0.65203300 |
| H | 6.83228700 | -1.53058600 | 1.14260500 |

**Model+py**

| | | | |
|---|---|---|---|
| Al | 1.03448500 | 3.19090200 | 0.91317800 |
| O | 2.19859300 | 2.96741400 | -0.38669000 |
| Si | 2.85542300 | 2.04154000 | -1.55817000 |
| O | 1.75134900 | 0.94534400 | -2.17841100 |
| Si | 1.02199900 | -0.51203600 | -1.96467000 |
| O | 1.98091200 | -1.73843900 | -2.52615700 |
| Si | 3.47167400 | -2.43733500 | -2.27733500 |
| O | 4.62808100 | -1.25701400 | -2.09923300 |
| Si | 5.30428700 | 0.00782500 | -1.25289800 |
| O | 5.88727500 | -0.52913200 | 0.20652200 |
| Si | 5.60796700 | -1.33258600 | 1.63656200 |
| O | 4.82909800 | -2.77507800 | 1.33274400 |
| Si | 3.38996900 | -3.37217600 | 0.75150800 |
| O | 3.41539300 | -3.38723700 | -0.91168700 |
| O | 2.19918800 | 0.28033400 | 1.65608300 |
| Si | 1.26142100 | -1.01655600 | 1.22998400 |
| O | 0.71267700 | -0.77122900 | -0.32956900 |
| O | -0.48264700 | 2.31083300 | 0.82022600 |
| Si | -1.75424800 | 1.46702800 | 0.30734900 |
| O | -1.97172200 | -0.02332900 | 0.99072900 |
| Si | -1.59366700 | -1.35286700 | 1.87819300 |
| O | -2.84817900 | -1.37131300 | 2.97956400 |
| Si | -4.44331100 | -1.08445700 | 2.48359100 |
| O | -4.72836800 | 0.46585800 | 1.93423500 |

| | | | |
|---|---|---|---|
| Si | -4.65653400 | 1.53571600 | 0.65441300 |
| O | -4.86536900 | 0.79422700 | -0.82222700 |
| Si | -4.81668900 | -0.26136800 | -2.10923200 |
| O | -3.32556500 | -0.21670600 | -2.90504100 |
| Si | -1.86605700 | -0.36004500 | -2.10561300 |
| O | -0.39165000 | -0.55150200 | -2.86144300 |
| Si | 3.45649900 | 0.74317900 | 2.68271400 |
| O | 4.67514500 | -0.40368800 | 2.64881000 |
| O | 2.13899100 | -2.41313200 | 1.28696000 |
| O | -0.00117300 | -1.15296800 | 2.32185900 |
| O | -1.63231800 | -2.81160200 | 1.08270700 |
| Si | -2.08642100 | -3.20860600 | -0.46829900 |
| O | -3.65114300 | -3.78339200 | -0.49209800 |
| Si | -5.02070100 | -2.85676000 | -0.23742300 |
| O | -4.86404100 | -2.10845800 | 1.24005300 |
| O | -2.06118400 | -1.77382000 | -1.28140400 |
| O | -1.68417200 | 1.08574700 | -1.30686200 |
| O | -3.16816100 | 2.30342500 | 0.63342100 |
| O | -5.17407300 | -1.76632000 | -1.48895800 |
| O | 4.16035800 | 1.17248700 | -0.96178300 |
| H | 3.19716100 | -4.75293300 | 1.24439800 |
| H | -5.29263500 | -1.33093700 | 3.67032900 |
| H | -5.71219700 | 2.55771600 | 0.84708700 |
| H | -5.83731900 | 0.10674500 | -3.11582100 |
| H | 3.31564300 | 2.88544400 | -2.69444600 |
| H | 3.80094500 | -3.28495600 | -3.44351000 |
| H | 6.90330200 | -1.62329000 | 2.29055100 |
| H | 6.44047600 | 0.54510000 | -2.03944100 |
| H | -6.20775300 | -3.74104200 | -0.22607500 |
| H | -1.18589900 | -4.23786900 | -1.03432500 |
| H | 3.98139700 | 2.04508100 | 2.18632900 |
| H | 2.95480200 | 0.80217500 | 4.08777400 |

| | | | |
|---|---|---|---|
| H | 1.61576700 | 3.40127100 | 2.37991300 |
| C | -0.94841300 | 5.38504000 | 0.59869400 |
| C | 1.17508700 | 5.91513500 | -0.23378600 |
| C | -1.43602900 | 6.65605400 | 0.26954300 |
| H | -1.58883800 | 4.60899900 | 1.04859300 |
| C | 0.75988700 | 7.20380500 | -0.58981500 |
| H | 2.19519300 | 5.54705900 | -0.42822600 |
| C | -0.56824900 | 7.58266000 | -0.33126000 |
| H | -2.48668400 | 6.90553900 | 0.47872300 |
| H | 1.47048200 | 7.89333300 | -1.06895000 |
| H | -0.92519000 | 8.58897200 | -0.60158000 |
| N | 0.33691700 | 5.02975600 | 0.35698300 |

**3a**

| | | | |
|---|---|---|---|
| Al | 0.51447800 | 0.36151300 | -1.10403300 |
| O | 0.47502600 | 1.70262900 | 0.02263100 |
| Si | -0.21437700 | 2.65220200 | 1.14753200 |
| O | -1.46184000 | 1.87544300 | 1.95659000 |
| Si | -3.01753000 | 1.35626200 | 1.89948000 |
| O | -4.05289200 | 2.54975100 | 2.40266200 |
| Si | -4.49333200 | 4.09849800 | 1.98133600 |
| O | -3.14468700 | 4.98203200 | 1.58193800 |
| Si | -1.83887500 | 5.35270500 | 0.61915000 |
| O | -2.36148500 | 5.84448100 | -0.88066100 |
| Si | -3.31572400 | 5.56361400 | -2.21388100 |
| O | -4.82883200 | 5.05310100 | -1.73930000 |
| Si | -5.64043100 | 3.82864400 | -0.95793100 |
| O | -5.54062500 | 4.04965200 | 0.68780100 |
| O | -2.33461500 | 2.01887500 | -1.87619500 |
| Si | -3.70230200 | 1.30961500 | -1.27393800 |
| O | -3.43108700 | 0.91832700 | 0.32609200 |
| O | -0.66616900 | -0.90690000 | -0.81471200 |

| | | | |
|---|---|---|---|
| Si | -1.64032300 | -1.96716200 | -0.09992000 |
| O | -3.19851300 | -2.00352600 | -0.67103600 |
| Si | -4.49605400 | -1.50776100 | -1.54373400 |
| O | -4.80201000 | -2.85755000 | -2.47770400 |
| Si | -4.76223300 | -4.40865200 | -1.79562200 |
| O | -3.25196800 | -4.89827900 | -1.28411100 |
| Si | -2.10214900 | -4.85769000 | -0.07277200 |
| O | -2.77771800 | -4.76355200 | 1.44714700 |
| Si | -3.71944400 | -4.38001000 | 2.76401200 |
| O | -3.36233700 | -2.84062100 | 3.36443900 |
| Si | -3.30478300 | -1.48715700 | 2.38581200 |
| O | -3.21039200 | 0.07590000 | 2.96044400 |
| Si | -1.74713300 | 2.98616100 | -3.11889300 |
| O | -2.61609800 | 4.41592500 | -3.19168800 |
| O | -4.97467900 | 2.36093600 | -1.37362000 |
| O | -4.07059100 | -0.03602900 | -2.19906800 |
| O | -5.88814000 | -1.19347300 | -0.69010800 |
| Si | -6.25057500 | -1.38422400 | 0.92212800 |
| O | -7.09041800 | -2.80365200 | 1.16955700 |
| Si | -6.42800400 | -4.33613100 | 1.05074100 |
| O | -5.76187800 | -4.49133200 | -0.46593900 |
| O | -4.78406900 | -1.53567600 | 1.65931700 |
| O | -1.90317800 | -1.65437200 | 1.51341600 |
| O | -1.09405100 | -3.54022900 | -0.27718800 |
| O | -5.30275000 | -4.53564700 | 2.26444900 |
| O | -0.86175900 | 4.03070300 | 0.43399500 |
| H | -7.06276700 | 3.85155500 | -1.36221800 |
| H | -5.22952300 | -5.33384000 | -2.85262100 |
| H | -1.29051400 | -6.09406500 | -0.17176100 |
| H | -3.46733900 | -5.32762300 | 3.87319300 |
| H | -1.93315700 | 2.29732600 | -4.43175000 |
| H | 0.76105400 | 3.06689600 | 2.19017800 |

| | | | |
|---|---|---|---|
| H | -5.17977100 | 4.72732200 | 3.13105700 |
| H | -3.46085200 | 6.82296900 | -2.97864100 |
| H | -1.10780500 | 6.48468400 | 1.23997400 |
| H | -7.50194800 | -5.33991900 | 1.22793000 |
| H | -7.06105200 | -0.24937100 | 1.41844800 |
| C | 5.40157300 | -1.37524500 | 0.60923700 |
| Ru | 4.63913200 | 0.02694000 | -0.55732100 |
| N | 5.55773900 | -2.66336200 | 0.14619300 |
| N | 5.93014600 | -1.34090500 | 1.87384800 |
| C | 6.28699400 | -3.53941900 | 1.08186200 |
| C | 6.41244500 | -2.66064700 | 2.33785800 |
| H | 7.45506900 | -2.58248300 | 2.71016200 |
| H | 5.78420000 | -3.02285200 | 3.18206100 |
| H | 7.27580000 | -3.82057200 | 0.65682500 |
| H | 5.71823500 | -4.47525900 | 1.26297900 |
| C | 5.95941700 | -0.25729700 | 2.81757400 |
| C | 7.12750600 | 0.54192600 | 2.91731400 |
| C | 7.15023300 | 1.56189500 | 3.88971100 |
| C | 6.06693500 | 1.79290800 | 4.76179000 |
| C | 4.92775600 | 0.97126200 | 4.63866000 |
| C | 4.85065000 | -0.06125600 | 3.68233800 |
| H | 8.04937000 | 2.19616700 | 3.96656300 |
| H | 4.06635600 | 1.13683200 | 5.30724500 |
| C | 5.09338000 | -3.18922800 | -1.11376100 |
| C | 3.82213500 | -3.82671400 | -1.17075800 |
| C | 3.38143500 | -4.32558500 | -2.41190900 |
| C | 4.17180200 | -4.24946600 | -3.57513400 |
| C | 5.46252400 | -3.69657000 | -3.46150300 |
| C | 5.95788300 | -3.18448900 | -2.24509900 |
| H | 2.38539600 | -4.79615200 | -2.46439300 |
| H | 6.12246000 | -3.67983600 | -4.34523400 |
| C | 4.42742700 | 1.43210900 | 0.62446800 |

| | | | |
|---|---|---|---|
| H | 4.67471400 | 1.37955800 | 1.70207100 |
| C | 3.91890700 | 2.70071900 | 0.14653700 |
| C | 3.72687400 | 3.81924200 | 0.99849100 |
| C | 3.57668400 | 2.82611200 | -1.23330600 |
| C | 3.20903300 | 5.01913300 | 0.49684500 |
| H | 3.99223400 | 3.71980600 | 2.06299100 |
| C | 3.04687700 | 4.02220200 | -1.74093500 |
| C | 2.86640100 | 5.11033200 | -0.86622800 |
| H | 3.06266000 | 5.88294500 | 1.16267300 |
| H | 2.76155500 | 4.11830800 | -2.79683500 |
| H | 2.44510200 | 6.04693500 | -1.26408900 |
| O | 3.78880400 | 1.67923800 | -1.95479900 |
| C | 3.72340700 | 1.68782500 | -3.43309100 |
| H | 2.75090200 | 2.16374800 | -3.68744600 |
| C | 4.89959800 | 2.47132200 | -4.01486600 |
| H | 4.93083400 | 3.51700900 | -3.65062500 |
| H | 5.85485600 | 1.98034600 | -3.74243900 |
| H | 4.80903400 | 2.49969400 | -5.12035000 |
| C | 3.69492400 | 0.23263300 | -3.87847400 |
| H | 3.59632100 | 0.19405300 | -4.98232700 |
| H | 4.63634900 | -0.28355300 | -3.59854200 |
| H | 2.83459300 | -0.30377800 | -3.43546200 |
| C | 2.97647300 | -4.06175100 | 0.06064700 |
| H | 1.89609400 | -3.97642400 | -0.16873800 |
| H | 3.14779500 | -5.09195600 | 0.44661300 |
| H | 3.20445300 | -3.35076800 | 0.87576200 |
| C | 7.39946500 | -2.74341000 | -2.14761200 |
| H | 8.01571000 | -3.53374800 | -1.66298700 |
| H | 7.82882700 | -2.57182200 | -3.15422400 |
| H | 7.51578400 | -1.80817200 | -1.56629300 |
| C | 3.64800200 | -4.74982300 | -4.90233700 |
| H | 2.99576300 | -3.98803300 | -5.38335200 |

| | | | |
|---|---|---|---|
| H | 4.47030600 | -4.97432300 | -5.61094400 |
| H | 3.03455400 | -5.66598000 | -4.78046600 |
| C | 3.59794500 | -0.89654500 | 3.55094200 |
| H | 3.11693800 | -0.75288800 | 2.55984300 |
| H | 3.80783200 | -1.98304000 | 3.64730200 |
| H | 2.85788700 | -0.62904700 | 4.32954600 |
| C | 8.30552500 | 0.32601800 | 1.99703700 |
| H | 8.72601500 | -0.69790800 | 2.09906500 |
| H | 8.01505800 | 0.44776800 | 0.93140400 |
| H | 9.11912300 | 1.04292400 | 2.22158200 |
| C | 6.14044400 | 2.86910300 | 5.82220900 |
| H | 6.62765400 | 2.48394100 | 6.74523500 |
| H | 6.73638500 | 3.73994400 | 5.48113400 |
| H | 5.13318600 | 3.23021300 | 6.11200400 |
| Cl | 6.75163100 | 0.56933300 | -1.42763800 |
| H | -0.31912700 | 3.28542000 | -2.81713600 |
| Cl | 2.39876200 | -0.88741200 | -0.42173700 |
| H | 0.72895100 | 0.73144600 | -2.63614100 |

**3b**

| | | | |
|---|---|---|---|
| Al | 0.72151900 | -0.47894700 | 1.04022700 |
| O | 0.33398800 | 0.84899200 | 2.08220400 |
| Si | -0.53301100 | 2.04050300 | 2.77419900 |
| O | -2.17207700 | 1.70518200 | 2.71419500 |
| Si | -3.51096100 | 1.62582400 | 1.76526400 |
| O | -4.31836100 | 3.07250700 | 1.78423200 |
| Si | -4.02145300 | 4.67663100 | 1.44387200 |
| O | -2.49585300 | 5.09225000 | 1.95559000 |
| Si | -0.83365300 | 5.06202400 | 1.86459100 |
| O | -0.32819700 | 5.68189700 | 0.40739400 |
| Si | -0.41008900 | 5.60064900 | -1.25437500 |
| O | -2.00400100 | 5.55157400 | -1.73623500 |

| | | | |
|---|---|---|---|
| Si | -3.41650200 | 4.67860800 | -1.66316300 |
| O | -4.15829500 | 4.93178600 | -0.19553600 |
| O | -0.77197700 | 1.91532800 | -1.01321700 |
| Si | -2.38540500 | 1.67720400 | -1.28151700 |
| O | -3.09936600 | 1.26640800 | 0.17336400 |
| O | -0.67349400 | -1.33468000 | 0.41464700 |
| Si | -2.13526300 | -2.01240500 | 0.35988300 |
| O | -3.08510600 | -1.60530800 | -0.93188600 |
| Si | -3.60687000 | -0.80709900 | -2.26926000 |
| O | -3.71540700 | -2.05092300 | -3.37664200 |
| Si | -4.42371400 | -3.53326000 | -2.96579400 |
| O | -3.57549400 | -4.40475100 | -1.82424000 |
| Si | -3.27045300 | -4.66453300 | -0.20408200 |
| O | -4.59571300 | -4.35450400 | 0.75639100 |
| Si | -5.94652000 | -3.68028200 | 1.45582700 |
| O | -5.56053900 | -2.28938800 | 2.33768700 |
| Si | -4.67933300 | -1.02825400 | 1.68681900 |
| O | -4.54584500 | 0.47284500 | 2.40017200 |
| Si | 0.59428800 | 2.60675600 | -1.71960900 |
| O | 0.39605700 | 4.26878900 | -1.82020200 |
| O | -3.08367700 | 3.05989600 | -1.85868100 |
| O | -2.57172700 | 0.48507000 | -2.44158700 |
| O | -5.11636800 | -0.10954700 | -2.20555300 |
| Si | -6.29871000 | -0.16241500 | -1.03812000 |
| O | -7.47502200 | -1.27792800 | -1.42855900 |
| Si | -7.25254800 | -2.93619400 | -1.37188200 |
| O | -5.94909200 | -3.30521300 | -2.33667100 |
| O | -5.50916700 | -0.72548700 | 0.29641800 |
| O | -3.10947800 | -1.57100900 | 1.63852100 |
| O | -2.02363600 | -3.68058300 | 0.31249100 |
| O | -7.02302300 | -3.39848300 | 0.21418200 |
| O | -0.28188300 | 3.50364500 | 1.99731400 |

| | | | |
|---|---|---|---|
| H | -4.32320700 | 5.12422500 | -2.74308600 |
| H | -4.49017000 | -4.32035200 | -4.21741700 |
| H | -2.86342300 | -6.07914400 | -0.03104900 |
| H | -6.55571800 | -4.62468800 | 2.41935300 |
| H | -0.17755300 | 2.18136000 | 4.21186500 |
| H | -5.02692100 | 5.50477500 | 2.14475900 |
| H | 0.21622400 | 6.81802200 | -1.81802900 |
| H | -0.29147000 | 5.92480800 | 2.94202300 |
| H | -8.46724400 | -3.60490700 | -1.89105200 |
| H | -6.93042700 | 1.16441700 | -0.86021200 |
| C | 5.52676200 | 0.04605200 | -0.67345400 |
| Ru | 3.58472500 | -0.32835800 | -0.16446100 |
| Cl | 3.09065600 | -0.49666600 | -2.47898900 |
| N | 5.94054600 | 1.32280700 | -0.97817000 |
| N | 6.56750400 | -0.78660000 | -1.01565800 |
| C | 7.23448100 | 1.36123600 | -1.69219100 |
| C | 7.76975000 | -0.05498300 | -1.47798500 |
| H | 8.56716700 | -0.10388700 | -0.70102700 |
| H | 8.17519100 | -0.51456100 | -2.40208200 |
| H | 7.88972700 | 2.14956700 | -1.26890500 |
| H | 7.07049100 | 1.59442800 | -2.76771600 |
| C | 6.74963000 | -2.19397200 | -0.78823100 |
| C | 7.18867600 | -2.64023700 | 0.48850800 |
| C | 7.44731900 | -4.01262200 | 0.66181600 |
| C | 7.29819000 | -4.94075600 | -0.39100600 |
| C | 6.87163600 | -4.45975400 | -1.64422700 |
| C | 6.59928900 | -3.09390000 | -1.87395000 |
| H | 7.77570300 | -4.36725000 | 1.65348500 |
| H | 6.74818500 | -5.16892500 | -2.48012000 |
| C | 5.29377100 | 2.58248300 | -0.70229000 |
| C | 4.56476400 | 3.25302600 | -1.72107800 |
| C | 4.01026700 | 4.51423600 | -1.41959100 |

| | | | |
|---|---|---|---|
| C | 4.18636700 | 5.13440100 | -0.16784800 |
| C | 4.98474100 | 4.47610300 | 0.78796900 |
| C | 5.56330900 | 3.21717000 | 0.54144300 |
| H | 3.42550700 | 5.02903600 | -2.19983700 |
| H | 5.17640600 | 4.96296100 | 1.75884900 |
| C | 3.79248500 | -2.10622300 | 0.29802500 |
| H | 4.78274000 | -2.54890600 | 0.07296500 |
| C | 2.86591700 | -3.16254000 | 0.73919400 |
| C | 3.11957700 | -4.46721700 | 0.21978000 |
| C | 1.75495500 | -3.05048300 | 1.62044200 |
| C | 2.29443900 | -5.56006400 | 0.50679200 |
| H | 3.99171100 | -4.59457000 | -0.44159500 |
| C | 0.91280100 | -4.13494300 | 1.90430600 |
| C | 1.17865900 | -5.39467300 | 1.34603200 |
| H | 2.51793900 | -6.54474400 | 0.06742400 |
| H | 0.03170700 | -3.98084300 | 2.54085000 |
| H | 0.50726700 | -6.23950000 | 1.56246900 |
| O | 1.46268400 | -1.80149100 | 2.21552600 |
| C | 4.43523700 | 2.69473300 | -3.11939300 |
| H | 3.59156700 | 3.17022000 | -3.65820300 |
| H | 5.35154900 | 2.90742500 | -3.71500500 |
| H | 4.26936900 | 1.59858000 | -3.12257700 |
| C | 6.47761600 | 2.58798200 | 1.56574800 |
| H | 6.67505600 | 3.29095700 | 2.39866000 |
| H | 6.01924300 | 1.67105900 | 1.99059400 |
| H | 7.45633600 | 2.30224100 | 1.12383600 |
| C | 3.53524300 | 6.46338700 | 0.13961600 |
| H | 2.47647500 | 6.32155600 | 0.44914400 |
| H | 4.05169300 | 6.99261400 | 0.96571000 |
| H | 3.52609200 | 7.13022700 | -0.74702500 |
| C | 6.13804200 | -2.61586400 | -3.23025300 |
| H | 5.20065500 | -2.02371400 | -3.15736100 |

| | | | |
|---|---|---|---|
| H | 6.89479600 | -1.95817900 | -3.71174300 |
| H | 5.96520800 | -3.47072100 | -3.91277600 |
| C | 7.32117300 | -1.67507000 | 1.64373100 |
| H | 8.02266900 | -0.84467100 | 1.41499400 |
| H | 6.34597500 | -1.20142900 | 1.88792000 |
| H | 7.69487000 | -2.18922100 | 2.55053000 |
| C | 7.61039300 | -6.40530700 | -0.17715700 |
| H | 8.70537200 | -6.56968900 | -0.07466000 |
| H | 7.14045900 | -6.79084200 | 0.75169200 |
| H | 7.25885100 | -7.02768700 | -1.02392000 |
| H | 1.73526000 | 2.29163100 | -0.81858600 |
| Cl | 3.64645700 | 0.74263200 | 1.94994500 |
| H | 0.75637800 | 2.10754600 | -3.11562500 |
| H | 1.78772400 | -0.41898400 | -0.17840600 |
| C | 1.76775200 | -1.55347300 | 3.68279600 |
| H | 1.83528200 | -0.44807400 | 3.68292700 |
| C | 3.11415400 | -2.15446100 | 4.04504900 |
| H | 3.91233100 | -1.74766000 | 3.39418100 |
| H | 3.34943300 | -1.87281300 | 5.09196900 |
| H | 3.10668000 | -3.26203400 | 3.98424500 |
| C | 0.59858500 | -2.01911600 | 4.54025800 |
| H | 0.74191600 | -1.63670200 | 5.57228900 |
| H | -0.36322200 | -1.61783000 | 4.16361200 |
| H | 0.53464200 | -3.12447500 | 4.60072100 |
